# Comparative proteomic study reveals the enhanced immune response with the blockade of interleukin 10 with anti-IL-10 and anti-IL-10 receptor antibodies in human U937 cells

**DOI:** 10.1371/journal.pone.0213813

**Published:** 2019-03-21

**Authors:** Guoying Ni, Shu Chen, Jianwei Yuan, Shelley F. Cavezza, Ming Q. Wei, Hejie Li, Xuan Pan, Xiaosong Liu, Tianfang Wang

**Affiliations:** 1 The First Affiliated Hospital/School of Clinical Medicine of Guangdong Pharmaceutical University, Guangzhou, China; 2 Genecology Research Centre, University of the Sunshine Coast, Maroochydore DC, Australia; 3 School of Medical Science, Griffith Health Institute, Griffith University, Gold Coast, Australia; 4 Cancer Research Institute, First People’s Hospital of Foshan, Foshan, Guangdong, China; 5 School of Health and Sport Sciences, University of the Sunshine Coast, Maroochydore DC, Australia; 6 Institute of Industrial Science, Department of Mechanical and Bio-functional System, the University of Tokyo, Tokyo, Japan; University of Toledo, UNITED STATES

## Abstract

Blocking cytokine interleukin 10 (IL-10) at the time of immunisation enhances vaccine induced T cell responses and improves control of tumour cell growth *in vivo*. However, the effect of an IL-10 blockade on the biological function of macrophages has not been explored. In the current paper, a macrophage precursor cell line, U937 cells, was selected to investigate the differential expression of proteins and relevant cell signalling pathway changes, when stimulated with lipopolysaccharide (LPS) in the presence of antibodies to IL-10 or IL-10 receptor. We used a quantitative proteomic strategy to investigate variations in protein profiles of U937 cells following the treatments with LPS, LPS plus human anti-IL10 antibody and anti-IL10R antibody in 24hrs, respectively. The LPS treatment significantly activated actin-related cell matrix formation and immune response pathways. The addition of anti-IL10 and anti-IL10R antibody further promoted the immune response and potentially effect macrophage survival through PI3K/AKT signalling; however, the latter appeared to also upregulated oncogene XRCC5 and Cajal body associated processes.

## Introduction

Cancer therapeutic vaccines are gradually becoming a cancer treatment modality, especially when combined with immune checkpoint inhibitors[1, 2]. Therapeutic vaccine induced T cells, especially CD8+ T cells, are able to destroy tumour cells without damaging nearby normal cells or tissues and cause less side effects compared with conventional surgery, chemo-or radiotherapy[3, 4]. Ideally, a therapeutic vaccine should elicit sufficient numbers of effector T cells, that can migrate to the tumour site, overcome the tumour suppressive microenvironment and kill the tumour cells. Tremendous efforts have been made to increase the efficacy of a therapeutic vaccine[5, 6].

Interleukin 10 (IL-10) is a cytokine with various biological functions[7] and is produced by many different myeloid and lymphoid cells. The primary function of IL-10 is to limit the amplitude of the immune response to avoid excessive immune activation to both self and foreign antigens that may result in autoimmune pathology or increased disease severity. IL-10 mediates its biological effects mainly through interaction with the IL-10 receptor on the membrane of professional antigen presenting cells and naïve and effector T cells[8].

We previously showed that temporal locking of IL-10 signalling at the time of immunisation drastically increases vaccine induced cytotoxic T cell responses compared with the same vaccine without IL-10 signalling blockade, and reduced tumour growth in an animal tumour model[4, 9]. HPV16 E7 long peptide-based vaccine with incomplete Freud adjuvants has been shown to be effective against the pre- cancerous condition, CIN3 in a clinical trial[10]. In an animal model blocking IL-10 at the time of E7 long peptide/MPLA immunisation prevents HPV16 E6/7 transformed TC-1 tumour growth[11]. This latter immunisation strategy was more efficacious in preventing tumour growth than immunisation without the IL-10 signalling blockade[12]. The underlying mechanism may include enhanced stimulation of dendritic cells by Toll Like Receptor (TLR) ligands, such as CpG orLPS, when IL-10 signalling is blocked. Generally, IL-10 signalling blockade is achieved through administration of anti-IL-10 or anti-IL-10 receptor mono-clonal antibodies. Therefore, only those cells with an IL-10 receptor on their membrane will be affected when IL-10 signalling is blocked.

Macrophage are innate immune cells which respond instantly to infection through TLRs. Tumour associated macrophages play both tumour promoting and anti-tumour roles depending on the tumour microenvironment. IL-10 can mediate an anti-inflammatory response in macrophages by metabolic reprogramming[13]. It has also been found that macrophage-derived IL-10 contributes to the control of the LPS response[14]. It is therefore of importance to investigate if and how the biological activities of macrophage are affected when stimulated by a TLR ligand simultaneously with an IL-10 signalling blockade.

U937 is a pro-monocytic, human myeloid leukaemia cell line and was isolated from the histiocytic lymphoma of a 37 year old male[15]. This cell line exhibits many characteristics of monocytes and has been widely used in the investigation of the mechanisms involved in monocyte-endothelium attachment[16]. They have also been used as the experimental model to elucidate mechanisms of monocyte and macrophage differentiation, and investigations into the tumorous nature of the cell line[17].

Our previous studies have shown that the use of antibodies to IL-10 and IL-10R downregulated the expression of IL-10 in several cell lines including U937[18, 19]. Here we report the molecular response at the protein level in the human monocytic (ML) cell line U937 stimulated with LPS when IL-10 signalling is blocked by anti-IL-10 or anti-IL-10R antibodies. The iTRAQ-labelling quantitative proteomic technique was used to compare for variation in protein abundance due to different treatments. The gene ontology enrichment and protein-protein interactions related to significantly regulated proteins were analysed. In addition, the modulation of biological signalling pathways was elucidated based on protein quantity.

## Materials and methods

### Chemicals

Trifluoroacetic acid (TFA), methanol, acetonitrile (ACN), formic acid (FA), NH_4_HCO_3_, urea, dithiothreitol (DTT), iodoacetamide (IAA), sodium pyruvate, L-glutamine, G418, lipopolysaccharide and non-essential amino acid solution were obtained from Sigma-Alderich (St. Louis, MO). Trypsin (Mass Spec grade V5280) was purchased from Promega (Madison, WI). Ultrapure water was prepared by Milli-Q water purification system (Millipore, Bedford, MA). Isobaric tag for relative and absolute quantitation (iTRAQ) 4-plex kit was purchased from AB SCIEX (Concord, Canada).

### Cell line, cell culture and antibodies

Human macrophage cell line U937 was purchased from Shanghai Institutes for Cell Resource Centre, Chinese Academy of Sciences, and cultured following the protocols in the product sheets. Briefly, U937 cells were maintained in complete RPMI 1640 media (Gibco) supplemented with 10% heat inactivated FCS, 100 U of penicillin/ml and 100 μg of streptomycin/ml and were cultured at 37°C with 5% CO_2_.

Anti-IL-10 receptor (1B1.3) monoclonal antibody (MAb) for *ex vivo* immunisation was purchased from BioXcell (USA) and stored at -80°C till further use. Anti-IL-10 (Cat. 506802), anti-IL-10R antibodies (Cat. 308802) for *in vitro* experiments were purchased from BioLedgend (Karrinyup, WA, Australia). PE conjugated anti-IL-10R antibody (Cat. 308803) was purchased from BioLegend.

### Cell lysis and iTRAQ labelling of peptides

Unstimulated U937 cells were used as reference and cultured overnight. U937 cells were either stimulated with 4×10^−3^ μM of LPS overnight (positive control) or treated with LPS plus 10 μg/mL concentration of anti-IL-10 or anti-IL-10R antibodies, respectively. After the treatment, cell pellets were washed with 1 mL of cold PBS and counted, then 1×10^6^ cells were lysed with 300 μl of lysis buffer (8M urea, 0.8M NH_4_HCO_3_, pH 8.0) supplemented with 10 μl of protease inhibitor cocktail (80-6501-23, GE Healthcare, Little Chalfont, UK) to prevent protein degradation. The samples were then sonicated for 30 min on ice, and then centrifuged at 12,000× g at 4°C for 15 min. The supernatants were collected, and protein concentration in the cell lysates was measured using the Pierce BCA protein assay kit on a NanoDrop 2000 (Thermo Fisher Scientific, Bremen, Germany). Then, 500 μg of proteins were reduced with 5 μl of 100 mM DTT for 1 h at 37°C, and subsequently alkylated with 20 μl of 100 mM IAA for 1 h at room temperature (RT) in the dark, followed by the incubation with the addition of 20 μL of 100 mM DTT at RT for 45 min. The urea concentration was reduced to below 2M by diluting the reaction mixture with MilliQ water, then the proteins digested with sequencing grade modified trypsin (Promega, Madison, WI) at 1:50 enzyme‐to‐substrate ratio. After 4 h of digestion at 37°C, samples were diluted 1:4 with 50 mM NH_4_HCO_3_, 1 mM CaCl_2_ and another aliquot of the same amount of trypsin was added to the samples and further incubated at room temperature overnight (~16 h).

The digested samples were then acidified with 10% FA to pH<3. Tryptic peptides were desalted on Sep-Pak C18 columns (Waters, Milford, MA) and dried using Speed-Vac concentrator (Thermo Fischer, Massachussets, USA). Peptides (100 μg) from three samples were dissolved in 30 μL of 0.5 M triethylammonium bicarbonate, pH 8.5 solution, and mixed with 1 units of iTRAQ reagent that was dissolved freshly in 70 μL of ethanol. Channel 114 was used for labelling the reference (unstimulated), 115 for labelling LPS stimulated cells, 116 and 117 for labelling LPS plus anti-IL-10 or anti-IL-10 receptor antibodies, respectively. After 1 h incubation at RT, 300 μL of water was added and incubated for 30 min at RT to stop the reaction. Peptides labelled by different iTRAQ reagents were then mixed and concentrated to ~200 μL, and were desalted on C18 SPE columns, dried and stored at -20 ^o^C. Biological triplicates were prepared using the same protocol.

### NanoLC tandem TripleTof MS/MS analyses

The iTRAQ labelled peptides were resuspended in 25 μL 0.1% FA in MilliQ water and analysed by LC-MS/MS on a Shimadzu Prominance Nano HPLC (Kyoto, Japan) coupled to a Triple Tof 5600 mass spectrometer (AB SCIEX, Concord, Canada) with a nano electrospray ion source, following a similar protocol reported elsewhere [20]. Briefly, ten microliters of each replicate were injected onto a trap column, followed by fractionation on a 150mm x 75μm 300SBC18, 3.5um nano HPLC column (Agilent Technologies, Australia). Linear gradients of 1–60% solvent B over 60 min at 300 nL/min flow rate, followed by a steeper gradient from 60% to 80% solvent B in 5 min were used for peptide elution. Solvent B was held at 80% for 5 min for washing the column and returned to 1% solvent B for equilibration prior to next sample injection. Solvent A consisted of 0.1% formic acid (aq) and solvent B contained 90/10 acetonitrile/ 0.1% formic acid (aq). The ionspray voltage was set to 2400V, declustering potential (DP) 100V, curtain gas flow 25, nebuliser gas 1 (GS1) 12 and interface heater at 150 ^o^C. The mass spectrometer acquired 500ms full scan TOF-MS data followed by 20 by 50 ms full scan product ion data in an IDA mode. Full scan TOFMS data was acquired over the mass range 350–1800 and for product ion ms/ms 100–1800 with a charge state of +2 to +5. The data was acquired and processed using Analyst TF 1.5.1 software (AB SCIEX, Concord, Canada). Biological triplicates were analysed in parallel.

### Protein identification and quantification

The LC-MS/MS data were imported to the PEAKS studio (Bioinformatics Solutions Inc., Waterloo, ON, Canada, version 7.0) with the assistance of MS Data Converter (Beta 1.3, http://sciex.com/software-downloads-x2110). The database of human proteome used for the analysis in this study was downloaded from Uniprot (http://www.uniprot.org/proteomes/UP000005640) containing 70,613 entries. *De novo* sequencing of peptides, database search and characterising specific PTMs were used to analyse the raw data; false discovery rate (FDR) was set to ≤ 1%, and [-10*log(p)] was calculated accordingly where p is the probability that an observed match is a random event. The PEAKS used the following parameters: (i) precursor ion mass tolerance, 0.1 Da; (ii) fragment ion mass tolerance, 0.1 Da (the error tolerance); (iii) tryptic enzyme specificity with two missed cleavages allowed; (iv) monoisotopic precursor mass and fragment ion mass; (v) a fixed modification of cysteine carbamidomethylation; and (vi) variable modifications including iTRAQ (for cell protein quantitation only), lysine acetylation, deamidation on asparagine and glutamine, oxidation of methionine and conversion of glutamic acid and glutamine to pyroglutamate.

For iTRAQ quantification, peptides with confidence ≥ 99% were used in ProteinPilot 5.0 software (AB SCIEX, Concord, Canada). The mass error tolerance was set to 0.1 Da, and the peptide score threshold (-10lgP) was set to that corresponding to 1% FDR. The results of differentially expressed proteins were validated sequentially by the following criteria, the proteins must contain at least one unique high confident peptide, the proteins have a P values < 0.05 and FDR ≤ 1%, and the fold change of proteins ≥ 1.5. To be less restrictive in the identification of proteins with altered relative abundance in treated cells with respect to the control (unstimulated) group, a protein was included in the analysis when it was confidently identified in at least two biological replicates.

### Gene ontology and pathway analysis

The significantly up- or down- regulated proteins were subjected to gene ontology and pathway analysis using the online tool ToppFunc[21], respectively. ToppFunc adopts a hypergeometric model to evaluate the annotation frequency of an input gene list with respect to the one that randomly occurs. Chromosome cytoband-based enrichment analysis was used to identify the genomic regions, where the input genes were significantly enriched using all the genes in these regions as background. Similar procedures were adopted to elucidate enriched gene ontology terms and pathways. In these enrichment analyses, all human genes in ToppFunc were used as background to calculate statistical significance. In addition, the Benjamini–Hochberg method implemented in ToppFunc was used to further exclude false negative results. A *P*-value <0.01 was adopted as the cutoff for enriched pathways and gene ontology.

### Protein-protein interaction (PPI) analysis

Interactions among significantly regulated proteins were predicted using STRING[22]. STRING provides a critical assessment and integration of protein-protein interactions from multiple resources, including direct (physical) as well as indirect (functional) associations. A spring model to generate the network images. All resources were selected to generate the network and ‘confidence’ was used as the meaning of network edges. Neither the 1^st^ nor 2^nd^ shell of the PPI was included in this study. Protein without any interaction with other proteins was excluded from the network of this study.

### Pathway analysis

The proteins determined to be differentially expressed were imported in Ingenuity Pathway Analysis (IPA; Qiagen, Redwood City, CA). In pathway analysis, the up-/down-regulation of key regulators identified in different treatments were used to predict the activation/inhibition of signalling pathways. Significance of the activation or inhibition of pathways predicted by the analysis was tested by the Fisher Exact Test *P* value, considering only the predictions with significant *P* value of <0.05 and a regulation z-score of <− 2 or >2 for inhibition and activation, respectively.

## Results

### Quantitative proteomic analysis reveals differential protein expression in U937 cells treated with LPS, LPS+anti-IL-10 or anti-IL-10 receptor antibodies

**Fig 1:**
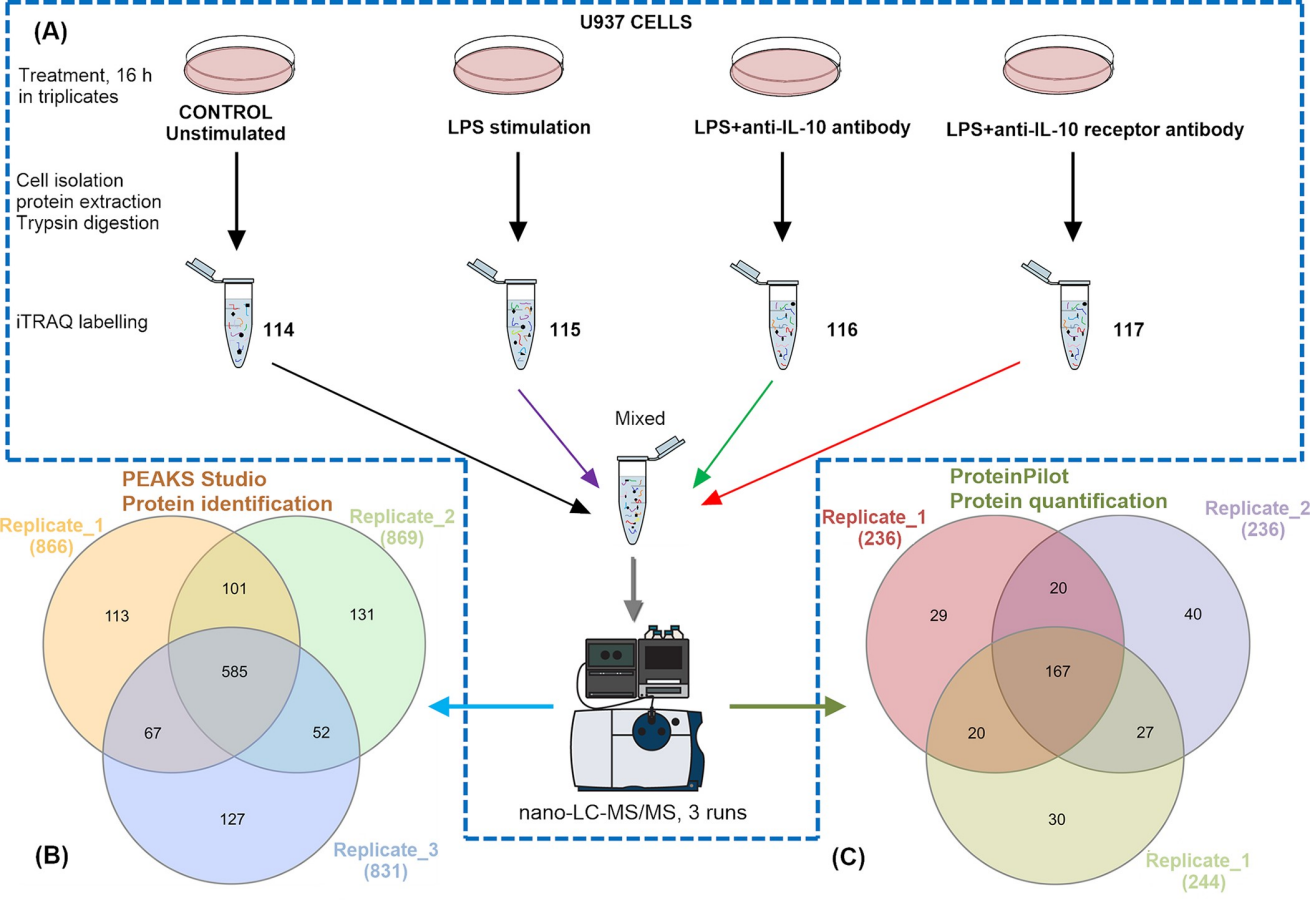
Overall workflow for quantitative proteomic analysis of U937 cells with different treatments, including LPS, LPS plus anti-IL-10 antibody and LPS plus anti-IL-10 receptor antibody. (A) Untreated and treated cell proteins/peptides were extracted and labelled with iTRAQ 4-plex reagent, followed by identification and quantification with high-accuracy nano-LC TripleTOF MS. (B) The comparison of proteins identified from three replicates (including proteins supported by both labelled and unlabelled peptides). (C) The comparison of quantifiable proteins in three replicates. See **S1** and **S2 Tables** for more details.

To reveal the molecular insights underlying the immunisation enhancement of blocking IL-10, iTRAQ 4-plex labelling in conjunction with nano-LC-MS/MS was used to assess the differential expression of the proteome of U937 cells. A workflow for the preparation and analysis is shown in **Fig 1A**. U937 cells were cultured for 16hrs with different treatments and untreated cells were used as controls. In total, iTRAQ analysis was used in twelve sets of experiments (biological triplicates), i.e., using 114 for labelling controls, 115 for LPS stimulation, 116 and 117 for LPS stimulation in conjunction with anti-IL-10 or anti-IL-10 receptor antibodies (**Fig 1A**). Similar initial amounts of extracted proteins were digested, labelled and mixed to generate three replicates for nano-LC-MS/MS analysis.

As shown in **Fig 1B**, there were 866, 869 and 831 proteins from 174, 186 and 190 protein groups identified in the triplicates, respectively, including both labelled and unlabelled proteins. The detailed information of protein identification is recorded in **S1 Table**. The comparison among the triplicates showed that 585 proteins were mutually identified (**Fig 1B**). After protein validation as described in Method, there were 236, 255 and 244 iTRAQ-labelled proteins detected in the samples with LPS stimulation, LPS plus anti-IL-10 antibody or anti-IL-10R antibody treatments, respectively (**S2 Table**). Of these quantifiable proteins, 234 proteins were identified in at least two replicates (**Fig 1C**). The fold-change values of these proteins with respect to untreated proteins and relevant protein annotations were shown in **S2 Table**. The hierarchy clustering result of quantitative analysis were displayed in **S1 Fig** Three replicates consistently showed that there were more significantly regulated proteins (fold-change greater than ±1.5) with LPS treatment only, than those with additional anti-IL10 or anti-IL10 antibody (**S2 Table**). In terms of gene oncology and pathway analysis, only those proteins showing significant quantitative variation consistently across biological triplicates were taken into consideration (inconsistency was highlighted in **S2 Table**); the accessions and the fold-change values of these proteins were also recorded.

### LPS treatment activated cell matrix formation and suppressed the signalling of Rho family proteins

**Fig 2:**
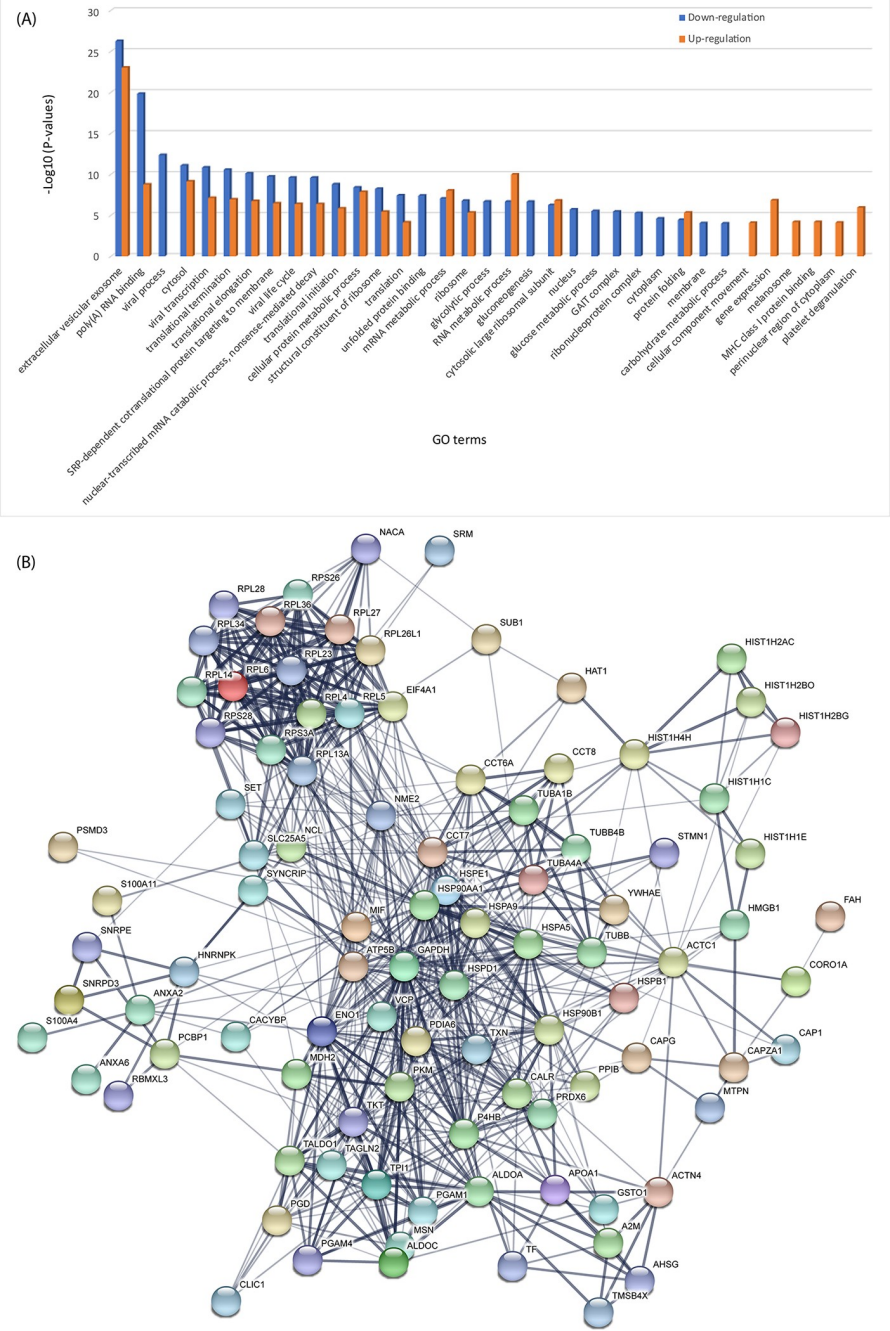
The gene ontology and protein-protein interaction (PPI) analysis of significantly regulated proteins identified in LPS-treated U937 cells by iTRAQ-label LC-MS/MS. (A) The GO terms enriched in significant up/down-regulated proteins; (B) The PPI network predicted based on significantly regulated proteins (see **S3 Table** for more details).

The gene oncology terms of significantly regulated proteins due to the LPS treatment were analysed with detailed information provided in **S3 Table**. **Fig 2A** compares enrichments of those proteins with corrected *P*-values lower than 10^−4^ (uncorrected *P*-values < 10^−5^). Similarities can be observed for the enrichments of GO terms between up- and down-regulated proteins, most of which origin from extracellular vesicular exosome and cytosol; they relate to viral transcription, translation, viral life cycle, SRP-dependent co-translational protein targeting to membrane, nuclear-transcribed mRNA catabolic process and so forth, with molecular functions of RNA binding and structural constituent of ribosome. The down-regulated proteins had lower *P*-values for these mutually regulated GOs. The viral process, unfolded protein binding, glycolytic, glucose and carbohydrate metabolic process were significantly suppressed, as they appeared to be more enriched in down-regulated proteins; while RNA metabolism, protein folding, cellular component movement, gene expression, MHC class I protein binding and platelet degranulation seemed more stimulated in U937 cells due to LPS treatment. The protein-protein interactions among the significantly regulated proteins were investigated, to assess the correlations among them (**S3 Table**).

**Fig 2B** shows the PPI network with a total of 99 nodes and 661 edges, which has significantly more interactions than expected (the expected number of edges are 218 using Lambda calculation[23]). There was a cluster of ribosomal proteins (e.g., RPL4, 5, 6, 14, 23 and 28 etc.) interacting with each other, and they interplay with other nodes mainly via EIF4A1, NME2, NCL and SLC25A5. The proteins with most interactions were mainly located in the central cluster of network, including heat shock proteins (HSP 10, 27, 60, 70 and 90), glyceraldehyde-3-phosphate dehydrogenase, valosin containing protein, protein disulphide isomerase family A, enolase 1, ATP synthase, thioredoxin, malate dehydrogenase 2, transketolase, prolyl 4-hydroxylase, aldolase A and peroxiredoxin 6, many of them are enzymes.

**Fig 3:**
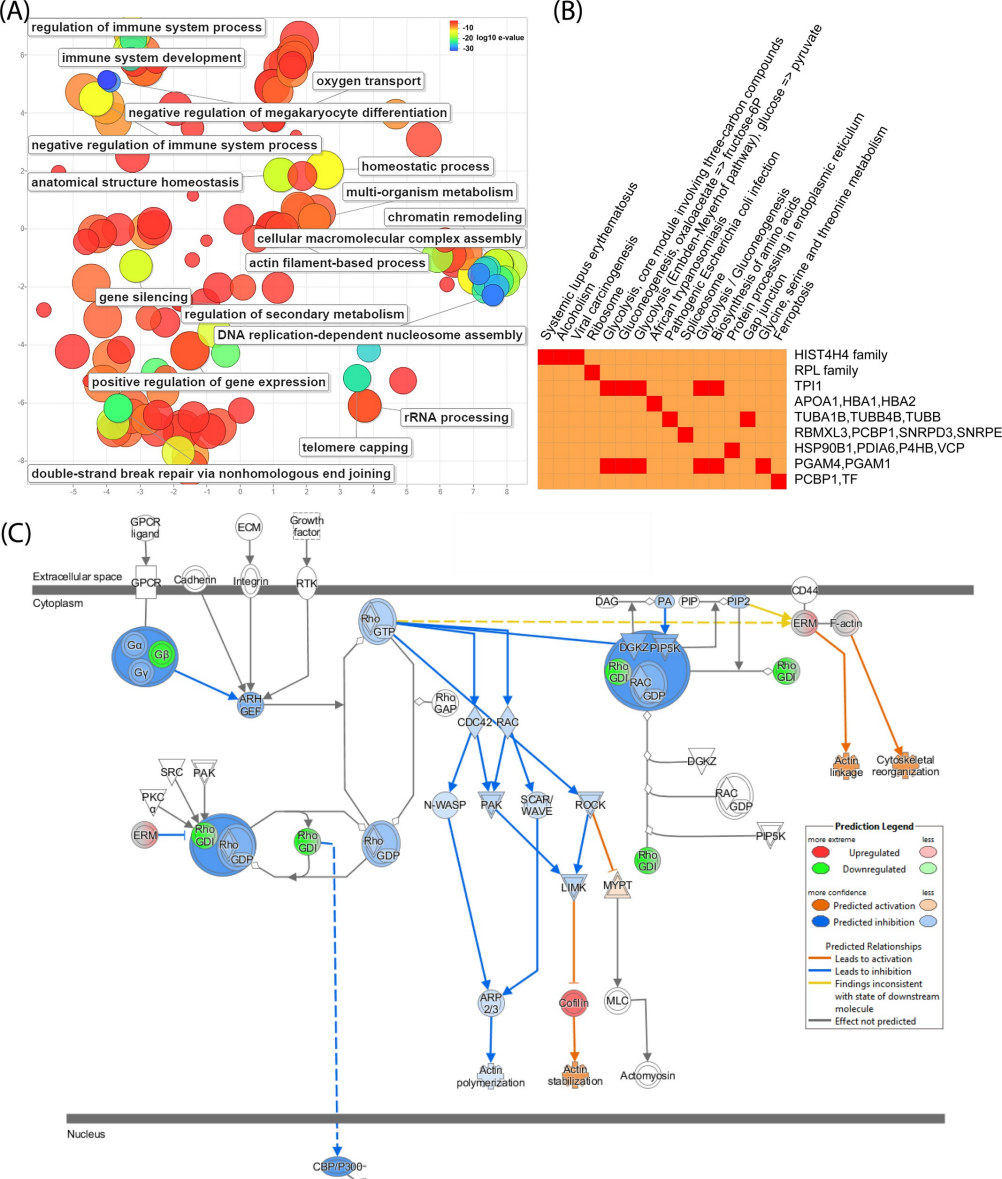
The significant biological processes and KEGG pathways identified from significantly upregulated proteins with LPS treatment. RhoGDI family signalling pathway was predicted by IPA as the canonical pathway with the highest predicted potential/significance. (A) The significant biological processes with corrected P-values<10^−6^; (B) The KEGG pathways with corrected P-values<0.05 and (C) The up-/down-regulations of proteins induced by LPS are indicated by nodes with a range of red and green intensities. The activation or inhibition of certain signalling was shown in orange or blue, respectively. Lines connecting the proteins represent known interactions, and arrows indicate the directions of up-/down-stream regulations.

The GO enrichment of upregulated proteins was further investigated in terms of biological processes (**Fig 3A**). There are 38 genes actively involved in negatively regulating megakaryocyte differentiation (GO:0045653, corrected *P*-value = 5.85E-33). We found 19 and 18 genes with differential expressions that play roles in the regulation of immune system development (GO:0002520, corrected *P*-value = 4.61E-11) and regulation of immune system process (GO:0002682, corrected *P*-value = 5.17E-7), respectively (**S4 Table**). There were 16 genes highly associate with negative regulation of immune system process (GO:0002683, corrected *P*-value = 1.53E-13). A total of 15 genes involved in DNA replication-dependent nucleosome organisation and assembly were identified with a corrected *P*-value of 2.54E-30. Besides, nucleosome and chromatin related pathways are also enriched (**S3 Table**). There were 16 KEGG pathways enriched post the treatment with the corrected *P*-values below 1.44E-2, such as systemic lupus erythematosus, alcoholism, viral carcinogenesis, ribosome, glycolysis and core module involving three-carbon compounds, etc. (**Fig 3B**).

The integrative pathway analysis revealed that the RhoGDI, EIF2, death receptor and 14-3-3 signalling pathways were significantly modulated. **Fig 3C** shows that the downregulation of Rho family proteins leads to the inhibition of Rho-GDP interaction, which consequently suppresses downstream regulators, including CDC42, ROCK and RAC, as well as the interactions among DGKZ, PIP5K, RhoGDI, RAC and GDP. Eventually, the polymerisation of actin in cytoplasm is inhibited due to the downregulation of ARP2/3. However, as the contents of cofilin, ERM and F-actin are elevated, the stabilisation and linkage of action and cytoskeletal reorganisation are therefore activated. Within the nucleus, the inhibition of CBP/P300 is suggested. Other three pathways are shown in **S1 File**. On EIF2 signalling pathway, many elongation factors are activated, thus the translation is enhanced. Because of the elevated level of 14-3-3 protein, ERK1/2, p90RSK and GSK3 are activated on the 14-3-3 mediated pathway, which causes the upregulation of TAU; in the nucleus, BAX and ELK1 are activated. In terms of death receptor signalling, CASP3, 6, 9 and 7 were inhibited yet the DNA repair was enhanced, leading to the suppression of cell apoptosis. Cell shrinkage is activated due to the upregulation of actin.

### Anti-IL-10 antibody enhanced immune response via inducing the expression of more heat shock proteins (HSPs)

**Fig 4:**
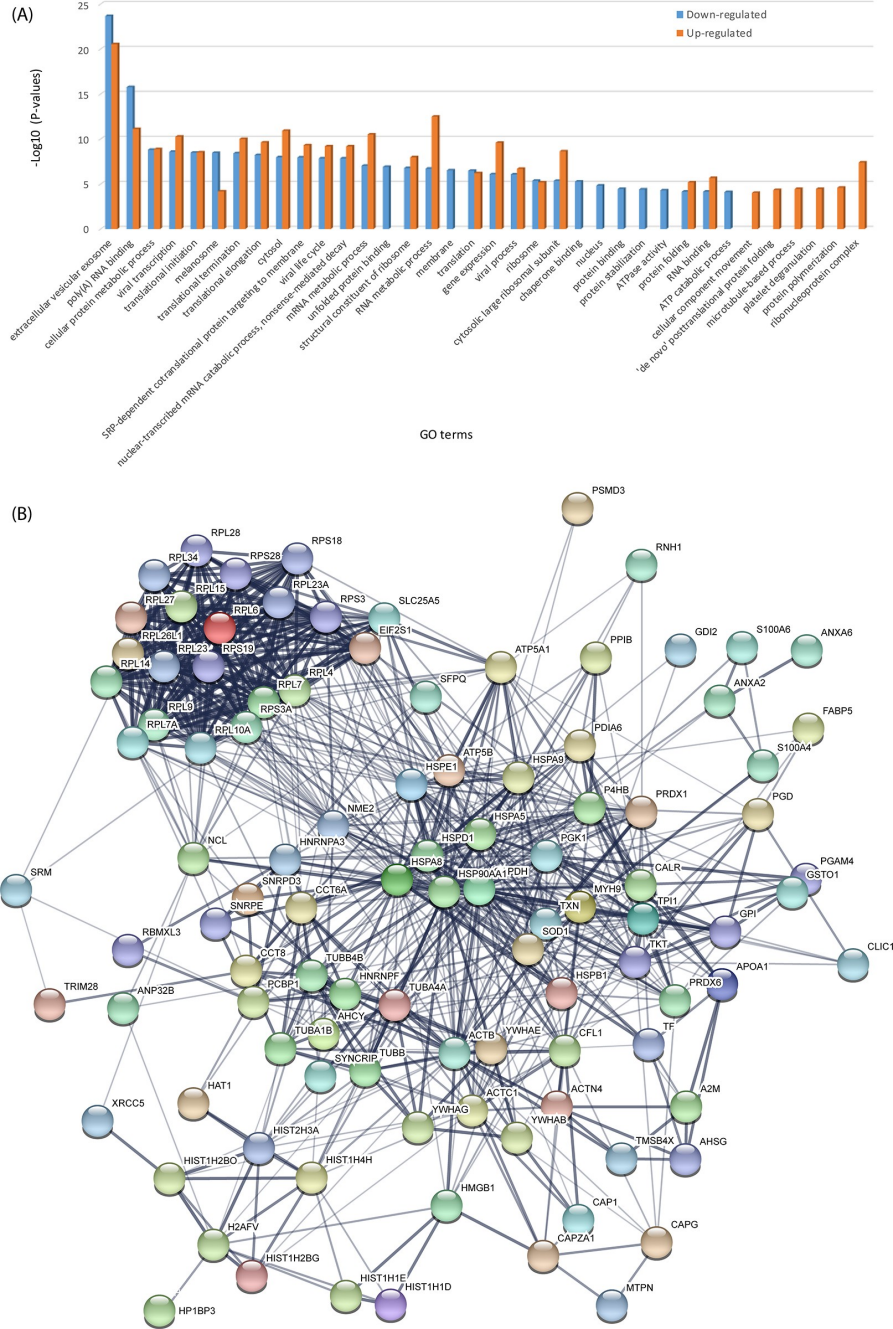
The gene ontology and protein-protein interaction (PPI) analysis of significantly regulated proteins identified in LPS+anti-IL10 antibody treated U937 cells by iTRAQ-labelled LC-MS/MS. (A) The GO terms enriched in significant up/down-regulated proteins; (B) The PPI network predicted based on significantly regulated proteins (see **S3 Table** for more details).

The GO enrichments of proteins with significant regulations with LPS in conjunction of anti-IL10 antibody treatment were comparatively displayed in **Fig 4A**, with details of GO information listed in **S3 Table**. It can be found that the mutually enriched terms were more significant in up-regulated protein group, which were different from those treated by LPS only (**Fig 3A**). The extracellular vesicular exosome and cytosol were the top two most enriched cellular component terms, similar to that of LPS treatment only. Chaperone binding, protein binding and stabilisation, ATPase activity, RNA binding and ATP catabolic process were more significantly suppressed; *de novo* posttranslational protein folding, microtubule-based process and protein polymerization appeared to be enhanced. The glycolytic, gluconeogenesis and carbohydrate metabolic processes became less significant with the addition of anti-IL10 antibody.

The significantly regulated proteins interact in a way displayed in **Fig 4B**, where 97 nodes and 661 edges can be determined; it has a *P*-value less than 10^−16^ with respect to whole human proteome, indicative of intensively interacting among them. Like the PPIs with LPS treatment only, there is a large cluster of ribosomal proteins, yet there are more S subunits present with additional anti-IL10 antibody treatment. Besides many interactions related to heat shock protein family, there are tyrosine 3-monooxygenase/tryptophan 5-monooxygenase activation protein (genes: *YWHAE* and *YWHAB*), cofilin 1 (*CFL1*), Superoxide dismutase 1 (*SOD1*), peroxiredoxin 1 (*PRDX1*), phosphoglycerate kinase 1 (*PGK1*) and myosin (*MYH9*) showing substantial number of interactions, and these nodes are not present in **Fig 3B**.

A total of 142 biological processes (**S2A Fig**) and 114 pathways (**S2B Fig**) are mutually identified as potentially enriched correlated with the significant protein upregulations, respectively. The enriched GO terms of significant up-regulated proteins (**S2C Fig**) show that there were more immune response related biological processes present (**S4 Table**) compared to LPS treatment, including innate immune response in mucosa (corrected *P*-value = 3.16E-10), mucosal immune response (corrected *P*-value = 4.40E-9), organ or tissue specific immune response (corrected *P*-value = 6.21E-9), innate immune response (corrected *P*-value = 1.61E-5), humoral immune response (corrected *P*-value = 2.23E-5), immune response (corrected *P*-value = 2.18E-3) and defence response (corrected *P*-value = 3.42E-3), which potentially resulted from the elevation of HSPs. Furthermore, three biological processes in response to bacteria were significantly detected, including antibacterial humoral response, defence response to Gram-positive bacterium and defence response to bacterium (**S4 Table**), which were not present with LPS only treatment. There were two more HSP related processes that emerge with the anti-IL10 antibody treatment, i.e., chaperone-mediated protein transport involved in chaperone-mediated autophagy and autophagy translocation complex disassembly. More RNA relevant processes, such as regulation of mRNA stability, RNA replication, viral RNA genome replication and regulation of RNA stability, became significant. Negatively regulations of tumour necrosis factor superfamily member 11 production and supermolecular fibre organisation seems to be additionally activated by anti-IL10 antibody (**S4 Table**). There were many metabolic processes that became less significant, such as NADH, NAD, ATP, NADP and a few pyridine and purine relevant processes. In addition, five actin-involved processes reduced their significance, including organisations of filament and cytoskeleton, filament-based process, rod assembly and filament capping.

**Fig 5:**
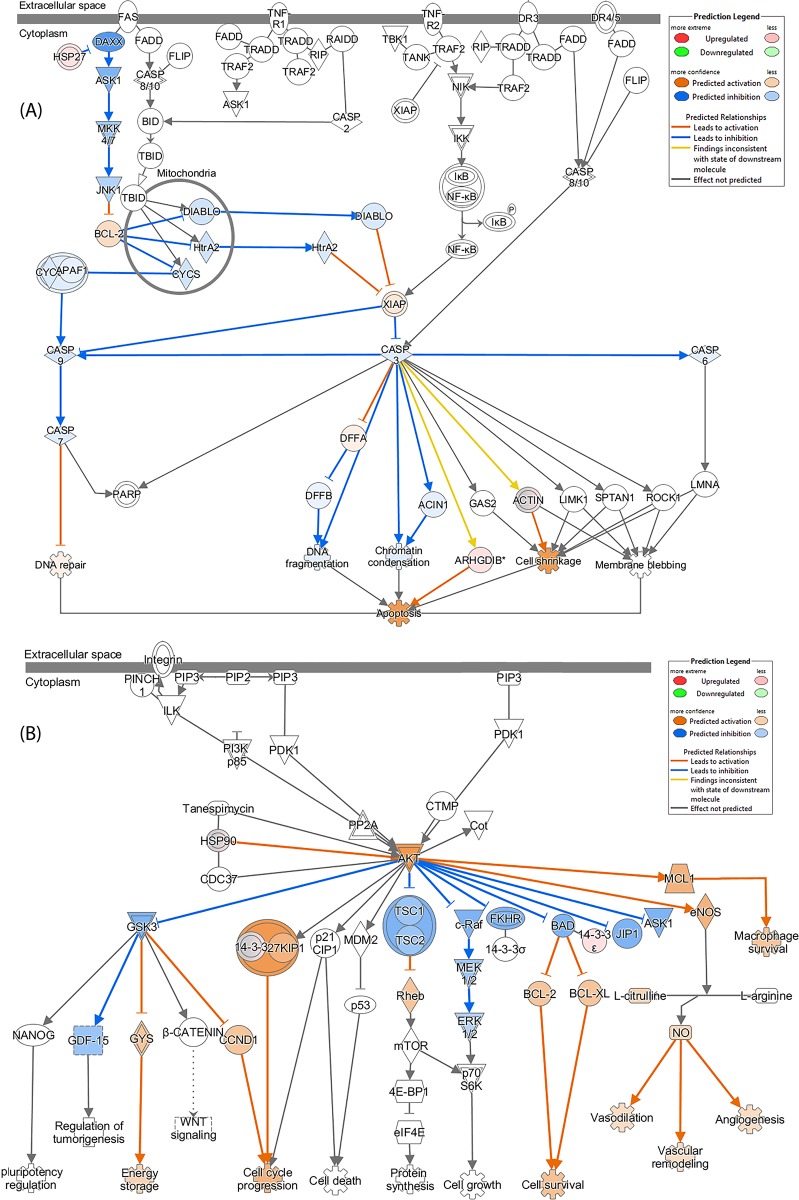
IPA identified the death receptor and PI3K/AKT signalling pathways as the canonical pathways in the U937 cells with the highest predicted potential/significance affected by the treatments with LPS+anti-IL10 and LPS+anti-IL10R antibodies, respectively, predicted based on iTRAQ-label quantitation. (A) the modulation of the death receptor signalling pathway by LPS+anti-IL10 antibody and (B) the modulation of the PI3K/AKT signalling pathway by LPS+anti-IL10R antibody. The up-/down-regulations of proteins are indicated by nodes with a range of red and green intensities. The activation or inhibition of certain signalling was shown in orange or blue, respectively. Lines connecting the proteins represent known interactions, and arrows indicate the directions of up-/down-stream regulations.

It appears that the anti-IL10 antibody significantly modulated the death receptor signalling (**Fig 5A**) compared to LPS only treatment (**S1 File**). This was mainly caused by the differential expression levels of ARHGDIB (Rho GDP-dissociation inhibitor 2), which showed downregulation in LPS only treatment, but upregulation with the presence of anti-IL10 antibody. As a result, apoptosis process was more activated with the addition of anti-IL10 antibody. The 14-3-3 signalling pathway was modulated in a comparable manner to LPS only treatment, which thus activated BAK and ELK1 within the nucleus (**S1 File**); 14-3-3 protein content was more elevated, and the higher abundance of PLC activated DAG. These two factors also contributed to the modulation of protein kinase A signalling (**S1 File**), where PKAr and PKAc were activated, thus cytoskeletal regulation was suppressed, yet histone H1 and H3B were upregulated in nucleus.

### Anti-IL-10R antibody modulated PI3K/AKT signalling to potentially effect cell survival

There were 43 proteins quantified as significantly down-regulated, while only two GO terms were found be enriched with anti-IL-10R antibody, i.e., extracellular vesicular exosome and RNA binding, as shown in **S3A Fig** This was remarkably different from those of LPS plus anti-IL10 antibody or LPS only treatments, respectively. Proteins involved in MHC class I protein binding process were enriched, in comparison to anti-IL10 antibody. The interactions of significantly regulated proteins were displayed in **S3B Fig** and the network information was kept in **S3 Table**. The number of nodes (76) and edges (390) were much less than those of U937 cells treated with LPS plus anti-IL10 antibody or LPS only, yet still representing intensive interactive network. The group of ribosomal proteins became less clustered due to less members identified, so were the central nodes that had less interactions identified. The HSPs, including HSP27, 70 and 90, were largely connected nodes though less than anti-IL10 antibody treatment. The nucleophosmin (*NPM1*), guanine nucleotide binding protein (*GNB2L1*) and cyclophilin A (*PPIA*) were newly identified as major nodes in this network, while VCP and MIF were missing in comparison with anti-IL10 treatment (**S3B Fig**).

LPS stimulated U937 cells treated with anti-IL10 or anti-IL10R antibody showed similar amounts of enriched biological processes, which were remarkably less than those of LPS only treatment (**S2A and S2B Fig**). There were several Cajal body relevant biological processes enriched uniquely due to proteins significantly upregulated with anti-IL10R antibody treatment compared to LPS plus anti-IL10 antibody or LPS only treatment, with positive regulation of protein (corrected *P*-value = 3.42E-4) and telomerase RNA localization to Cajal body (corrected *P*-value = 3.42E-4) being the most representative processes. In addition, protein localisations to nucleoplasm and nuclear body, protein refolding and formation of translation preinitiation complex processes were more representative in U937 cells treated with anti-IL10R than anti-IL10 antibody. The pathway analysis shown in **Fig 5B** suggests the PI3K/AKT signalling was significantly modulated, where AKT was activated by the upregulation of HSP90. None of other upstream regulator of AKT was largely influenced by the treatment. The downstream factors including MCL1, eNOS and 27KIP1 were accordingly enhanced, which in turn activated macrophage survival, production of NO and cell cycle progression (function in conjunction with 14-3-3), respectively. Other regulators, such as GSK3, FKHR, BAD, c-Raf and JIP1 etc., were suppressed. Consequently, the energy storage was activated because of GYS activation and the contribution of CCND1 to cell cycle progression; cell survival was suggestive with the upregulations of both BCL-2 and BCL-XL.

Previously, we demonstrated that blocking IL-10 signalling with an anti-IL10R antibody at the time of immunisation enhances vaccine-induced T cell responses [4]. We also demonstrated that LPS treated human PBMCs secreted more IL-12 in the presence of anti-IL-10 antibodies [19]. To further confirm that immune response would be enhanced by the application of anti-IL10R or anti-IL10 antibody, IL-6 or IL-12p40 were measured 24hrs post the treatments of U937 or mouse PBMCs (**S4 Fig**). The ELISA results clearly showed that IL-12p40 of PBMCs were significantly elevated by the addition of anti-IL10R or anti-IL10 antibody (**S4A Fig**), respectively; the enhanced level of IL-6 due to anti-IL10R was dose-dependent (**S4B Fig**). Significant more IL-12p40 was secreted by U937 cells treated by LPS plus 10μg anti-IL10R antibody (**S4C Fig**).

## Discussion

The iTRAQ labelling in conjunction of LC-MS/MS found that poly(rC) binding protein 1 (PCBP1) was significantly up-regulated with LPS treatment (see **S2 Table**). PCBP1 stimulates integral RNA entry segment (IRES)-mediated translation initiation *in vitro* and *in vivo*, where it functions as an RNA chaperone to open the RNA in the recruitment of ribosome[24]. It has been found that PCBP1 is induced under cell stress conditions via the mitogen-activated protein kinase signalling pathway[25], which has a positive correlation with the expression of HSP27 through the oxidative stress response[26]. The significantly upregulation of HSP27 detected aligned well with this pathway. Besides, other two HSP family members, including HSP70 and 90, were elevated compared to untreated cells. HSP70 plays role in the control of cell proliferation and cellular aging[27], while HSP90 contributes to not only processing and transporting secreted proteins[28], but also endoplasmic reticulum associated degradation[29] and relates to the folding of Toll-like receptors[30]. Thus, the elevated level of HSP90 aligned with the activation of Toll-like receptor due to LPS treatment[31].

There were several microfilament relevant proteins showing significant upregulation, such as tubulins and actinin, as well as additional proteins with the function of promoting the formation of filament, including myotrophin[32], capping protein (muscle Z-line, alpha 1) [33] and adenylate cyclase-associated protein 1[34], which suggests that the dynamics of filament was remarkably altered; this can also be predicted from the RhoGDI pathway modulation shown in **Fig 3C**. The interactions between herpesvirus and the actin cytoskeleton of the host cell and actin-regulating Rho GTPase play important roles in virus entry and replication[35], thus modulation of RhoGDI signalling may contribute to the interplay between LPS and the cell matrix. Besides HSP90 (with ATPase activity[36]) and ATP synthase, ATP related proteins showed up-regulation, for example, proteasome 26S subunit acts a regulator in the ATP-dependent degradation of ubiquitinated proteins[37]; valosin containing protein is involved in the formation of the transitional endoplasmic reticulum, which generates vesicle bubbles with the assistance of ATP[38]; and chaperonin containing TCP1 guides protein folding post ATP hydrolysis[39]. Together this suggest that the energy consumption within the cells increased as more depletion of ATP occurred due to the LPS treatment, which is also supported by the enriched glycolysis and other energy production KEGG pathways (see **Fig 3B**).

Glutathione transferase Omega 1 (GSTO1) were significantly upregulated in three treatments (see **S2 Table**). It has recently reported that GSTO1 plays a critical role in the pro-inflammatory response of macrophages to LPS[40, 41]; in addition, the genetic knockout of *GSTO1* in a mouse model remarkably reduced the pro-inflammatory response in three inflammatory disease models[42]. Our study strongly suggests that gene expression of *GSTO1* might also positively correlate with pro-inflammation in U937 cells stimulated by LPS, and the use of anti-IL-10 and anti-IL-10R antibodies were not able to influence this correlation.

The upregulation of Rho GDP-dissociation inhibitor 2 (ARHGDIB), known to play crucial role in the regulation of the GDP/GTP exchange reaction [43], was observed in U937 cells in the presence of anti-IL-10 or anti-IL-10R antibody (see **S2 Table**). ARHGDIB was found to show significant overexpression in COS-7 cells with the infection of enteropathogenic *E*. *coli*[44]. It has been reported that elevated ARHGDIB was able to attenuate HIV-1 infection via negatively regulating the replication of HIV-1[45]. Its upregulation was also identified in the response of the mouse nervous system to alpha herpesvirus infection at the latency and reactivation stages[46, 47], while its downregulation appears to be associated with herpesvirus entry and replication[48]. A recent report indicated that ARHGDIB produced by apoptotic T-cells can inhibit the growth of *Mycobacterium tuberculosis* and enhance the apoptosis of *M*. *tuberculosis*-infected human macrophages[49]. The addition of recombinant ARHGDIB to monocyte-derived macrophages resulted in the reduction of IL-10 secreted into the supernatant[49]. Our study suggested that additional anti-IL-10 or anti-IL-10R antibody further activated the pathway to produce more ARHGDIB in response to LPS stimulation.

Significant elevated level of 14-3-3 protein was only detected in the cells treated with additional anti-IL-10 antibody (see **S1 File**), which contributed to the presence of YWHAE and YWHAB on the PPI (see **Fig 4B**), suggesting tyrosine 3-monooxygenase/tryptophan 5-monooxygenase signalling was potentially activated[50]. 14-3-3 protein was found to negatively affect parainfluenza virus particle formation through interacting with PIV5M protein to reduce the budding of virus[51]. Also, it has been found that 14-3-3 protein is able to regulate the host inflammatory response to viral and bacterial infections through interaction with Toll-like receptors[52–54]. Thus, the addition of anti-IL-10 antibody could induce U937 cells to generate more immune response towards LPS stimulation via expressing higher concentration of 14-3-3 proteins.

PI3K/AKT pathway has been found to signal with multiple receptors, such as insulin receptors[55], pathogen recognition receptors[56] and cytokine receptors[57], thus it regulates macrophage responses by modulating the activation phenotype[58, 59]. Previous studies also pointed out that this pathway plays critical roles in macrophage biology and inflammatory disease regulation, via the regulations of inflammatory cytokines[59], miRNAs[60], together with molecular functions including phagocytosis[61], autophagy[62], and cell metabolism[63]. In this study, significant activation of this pathway was identified with the treatment of anti-IL-10 or anti-IL-10R antibody led by the upregulation of AKT, compared to LPS treatment only (see **Fig 5B** and **S1 File**). Consequently, macrophage survival might be increased to eliminate pathogens through phagocytosis. Meanwhile, the polarisation of M1 and M2 might also be activated[64, 65].

The upregulations of histone cluster 1 and 2 were identified with significance in the treatment with anti-IL-10 or anti-IL-10R antibody. This protein family is one of the core components of the nucleosome and has important function in transcription regulation, DNA repair, DNA replication and chromosomal stability[66]. Thus, its upregulation suggests increased activity of the nucleosome, which is in accordance with the higher abundance of elongation factors detected by LC-MS/MS; as a response, there was elevated level of TRIM28 expressed in cells that functions as transcription suppressor[67].

XRCC5 was significantly upregulated only in the presence of anti-IL-10R antibody and downregulated remarkably in LPS only treatment. XRCC5 is an ATP-dependent DNA helicase mapped to chromosome 2q35 and has been suggested as a potential chronic obstructive pulmonary disease susceptibility gene[68]. Other studies reported that XRCC5 promoted colon cancer growth through modulation of COX-2 signalling[69]; potentially associated with the development of systemic lupus erythematosus[70]; and related to hyper proliferation and resistance to apoptosis, genomic instability, and tumorigenesis[71]. Furthermore, Cajal body relevant processes were enriched in U937 cells treated with LPS plus anti-IL-10R antibody. It has been reported that Cajal body formation was induced *in vitro* by the overexpression of telomerase[72] and a few Cajal body-localised small nucleolar RNP components showed significantly higher levels in cancer[73, 74], indicating Cajal body might relate to cancer progression. These studies suggest that the upregulation of XRCC5 and Cajal body related proteins observed in this study, caused by the addition of anti-IL-10R antibody, might lead to oncogenic phenotypes.

## Conclusions

In summary we demonstrated that U937 cells (a macrophage precursor cell) when stimulated with TLR ligand 4, LPS, in the presence of anti-IL-10 or anti-IL-10 receptor antibodies, activated more immune-related signalling pathways than without IL-10 signalling blockade. This outcome may not only lead to higher vaccine induced immune responses but may also change the function of tumour infiltrating macrophages within the tumour microenvironment, contributing to better tumour growth control. Both antibodies enhanced macrophage survival via PI3K/AKT signalling. However, the application of anti-IL-10R antibody requires further study, as its usage might activate oncogene *XRCC5*. Ongoing studies that analyse the function of tumour residential macrophage is warranted when immunisation and temporal IL-10 signalling blockade is used to treat cancer patients.

## Supporting information

S1 Fig Hierarchy cluster of differentially expressed proteins in U937 cells identified from iTRAQ analysis of three biological replicates treated with LPS, LPS+anti-IL-10 antibody and LPS+anti-IL-10R antibody at 24 h. The magnitudes of up-/down- regulation are indicated by colour change. The fold change data was listed in **S2 Table**.
(TIF)

S2 Fig The biological process and KEGG pathway enrichment due to the treatments of LPS, LPS+anti-IL-10 and LPS+anti-IL-10R antibody. (A) Comparison of enriched biological processes. (B) Comparison of enriched biological pathways. The statistically over-represented gene ontology terms of cells with treated by LPS+anti-IL-10 antibody (C) and LPS+anti-IL-10R antibody (D).
(TIF)

S3 Fig The gene ontology analysis of significantly regulated proteins identified in LPS+anti-IL-10R antibody treated U937 cells by iTRAQ-label LC-MS/MS. (TIF)

S4 Fig 5×10^5^ of mouse PBMCs (A and B) or 5×10^5^ U937 cells (C) were either left untreated (UN) or stimulated with 100 ng of LPS, in the presence or absence of anti-IL-10 or anti-IL10R antibodies and cultured overnight. Supernatants were collected and measured by IL-6 or IL-12p40 ELISA kit from eBioscience (San Diego, CA, USA). Statistical analysis was performed by the two-tailed t-test.
(TIF)

S1 Table Protein identification results of three biological replicates of U937 cells treated by LPS, LPS+anti-IL-10 antibody and LPS+anti-IL-10R antibody. For each replicate, there are protein identified and supporting peptides, iTRAQ quantified proteins and *de novo* only peptides with average local confidence greater than 80%.
(XLSX)

S2 Table Protein quantitation results of three biological replicates of U937 cells treated by LPS, LPS+anti-IL-10 antibody and LPS+anti-IL-10R antibody, with respect to the control. For each replicate, there are all iTRAQ labelled proteins and significantly regulated proteins.
(XLSX)

S3 Table The gene oncology and PPIs of significantly regulated proteins. (XLSX)

S4 Table The over-represented biological processes and KEGG enriched corresponding to upregulated proteins of each treatment. (XLSX)

S1 File Other significant modulated canonical pathways identified from differentially expressed proteins identified from U937 cells with the treatments of LPS, LPS+anti-IL-10 antibody and LPS+anti-IL-10R antibody. (PDF)

## References

[1] Massarelli E, William W, Johnson F, Kies M, Ferrarotto R, Guo M, et al Combining Immune Checkpoint Blockade and Tumor-Specific Vaccine for Patients With Incurable Human Papillomavirus 16-Related Cancer: A Phase 2 Clinical Trial. JAMA Oncol. 2018 10.1001/jamaoncol.2018.4051 . 30267032 PMC6439768

[2] Dammeijer F, Lau SP, van Eijck CHJ, van der Burg SH, Aerts J. Rationally combining immunotherapies to improve efficacy of immune checkpoint blockade in solid tumors. Cytokine Growth Factor Rev. 2017;36:5–15. 10.1016/j.cytogfr.2017.06.011 . 28693973

[3] Chen J, Ni G, Liu XS. Papillomavirus virus like particle-based therapeutic vaccine against human papillomavirus infection related diseases: immunological problems and future directions. Cellular immunology. 2011;269(1):5–9. 10.1016/j.cellimm.2011.03.003 . 21477796

[4] Ni G, Wang T, Walton S, Zhu B, Chen S, Wu X, et al Manipulating IL-10 signalling blockade for better immunotherapy. Cellular immunology. 2015. 10.1016/j.cellimm.2014.12.012 25596475

[5] Kim J, Li WA, Choi Y, Lewin SA, Verbeke CS, Dranoff G, et al Injectable, spontaneously assembling, inorganic scaffolds modulate immune cells in vivo and increase vaccine efficacy. Nature biotechnology. 2015;33(1):64–72. Epub 2014/12/09. 10.1038/nbt.3071 25485616 PMC4318563

[6] Michel ML, Deng Q, Mancini-Bourgine M. Therapeutic vaccines and immune-based therapies for the treatment of chronic hepatitis B: perspectives and challenges. Journal of hepatology. 2011;54(6):1286–96. Epub 2011/01/18. 10.1016/j.jhep.2010.12.031 . 21238516

[7] Howard M, O'Garra A, Ishida H, de Waal Malefyt R, de Vries J. Biological properties of interleukin 10. Journal of clinical immunology. 1992;12(4):239–47. . 1512298 10.1007/BF00918147

[8] Couper KN, Blount DG, Riley EM. IL-10: the master regulator of immunity to infection. Journal of immunology (Baltimore, Md: 1950). 2008;180(9):5771–7. Epub 2008/04/22. . 18424693 10.4049/jimmunol.180.9.5771

[9] Liu XS, Leerberg J, MacDonald K, Leggatt GR, Frazer IH. IFN-gamma promotes generation of IL-10 secreting CD4+ T cells that suppress generation of CD8 responses in an antigen-experienced host. Journal of immunology. 2009;183(1):51–8. Epub 2009/06/19. 10.4049/jimmunol.0802047 . 19535638

[10] de Vos van Steenwijk PJ, Ramwadhdoebe TH, Lowik MJ, van der Minne CE, Berends-van der Meer DM, Fathers LM, et al A placebo-controlled randomized HPV16 synthetic long-peptide vaccination study in women with high-grade cervical squamous intraepithelial lesions. Cancer immunology, immunotherapy: CII. 2012;61(9):1485–92. 10.1007/s00262-012-1292-7 22684521 PMC3427705

[11] Ni G, Liao Z, Chen S, Wang T, Yuan J, Pan X, et al Blocking IL-10 signalling at the time of immunization does not increase unwanted side effects in mice. BMC immunology. 2017;18(1):40 Epub 2017/08/16. 10.1186/s12865-017-0224-x 28810829 PMC5557397

[12] Llopiz D, Aranda F, Diaz-Valdes N, Ruiz M, Infante S, Belsue V, et al Vaccine-induced but not tumor-derived Interleukin-10 dictates the efficacy of Interleukin-10 blockade in therapeutic vaccination. Oncoimmunology. 2016;5(2):e1075113 10.1080/2162402X.2015.1075113 27057445 PMC4801433

[13] Ip WKE, Hoshi N, Shouval DS, Snapper S, Medzhitov R. Anti-inflammatory effect of IL-10 mediated by metabolic reprogramming of macrophages. Science. 2017;356(6337):513–9. 10.1126/science.aal3535 . 28473584 PMC6260791

[14] Siewe L, Bollati-Fogolin M, Wickenhauser C, Krieg T, Muller W, Roers A. Interleukin-10 derived from macrophages and/or neutrophils regulates the inflammatory response to LPS but not the response to CpG DNA. European journal of immunology. 2006;36(12):3248–55. Epub 2006/11/18. 10.1002/eji.200636012 . 17111348

[15] Sundström C, Nilsson K. Establishment and characterization of a human histiocytic lymphoma cell line (U‐937). International journal of cancer. 1976;17(5):565–77. 178611 10.1002/ijc.2910170504

[16] Liu L, Zubik L, Collins FW, Marko M, Meydani M. The antiatherogenic potential of oat phenolic compounds. Atherosclerosis. 2004;175(1):39–49. Epub 2004/06/10. 10.1016/j.atherosclerosis.2004.01.044 . 15186945

[17] Strefford JC, Foot NJ, Chaplin T, Neat MJ, Oliver RT, Young BD, et al The characterisation of the lymphoma cell line U937, using comparative genomic hybridisation and multi-plex FISH. Cytogenetics and cell genetics. 2001;94(1–2):9–14. Epub 2001/11/10. 10.1159/000048774 . 11701946

[18] Ni G, Wang Y, Cummins S, Walton S, Mounsey K, Liu X, et al Inhibitory mechanism of peptides with a repeating hydrophobic and hydrophilic residue pattern on interleukin-10. Human vaccines & immunotherapeutics. 2017;13(3):518–27. Epub 2016/10/01. 10.1080/21645515.2016.1238537 27686406 PMC5360125

[19] Ni G, Chen S, Yang Y, Cummins SF, Zhan J, Li Z, et al Investigation the Possibility of Using Peptides with a Helical Repeating Pattern of Hydro-Phobic and Hydrophilic Residues to Inhibit IL-10. PloS one. 2016;11(4):e0153939 Epub 2016/04/23. 10.1371/journal.pone.0153939 27100390 PMC4839630

[20] Ni G, Liang D, Cummins SF, Walton SF, Chen S, Wang Y, et al Comparative Proteomic Study of the Antiproliferative Activity of Frog Host-Defence Peptide Caerin 1.9 and Its Additive Effect with Caerin 1.1 on TC-1 Cells Transformed with HPV16 E6 and E7. BioMed research international. 2018;2018:7382351 Epub 2018/06/05. 10.1155/2018/7382351 29862288 PMC5971270

[21] Chen J, Bardes EE, Aronow BJ, Jegga AG. ToppGene Suite for gene list enrichment analysis and candidate gene prioritization. Nucleic acids research. 2009;37(Web Server issue):W305–11. Epub 2009/05/26. 10.1093/nar/gkp427 19465376 PMC2703978

[22] Szklarczyk D, Franceschini A, Wyder S, Forslund K, Heller D, Huerta-Cepas J, et al STRING v10: protein-protein interaction networks, integrated over the tree of life. Nucleic acids research. 2015;43(Database issue):D447–52. Epub 2014/10/30. 10.1093/nar/gku1003 25352553 PMC4383874

[23] Hillenmeyer S, Davis LK, Gamazon ER, Cook EH, Cox NJ, Altman RB. STAMS: STRING-assisted module search for genome wide association studies and application to autism. Bioinformatics (Oxford, England). 2016;32(24):3815–22. Epub 2016/08/21. 10.1093/bioinformatics/btw530 27542772 PMC5167061

[24] Pickering BM, Mitchell SA, Spriggs KA, Stoneley M, Willis AE. Bag-1 internal ribosome entry segment activity is promoted by structural changes mediated by poly(rC) binding protein 1 and recruitment of polypyrimidine tract binding protein 1. Molecular and cellular biology. 2004;24(12):5595–605. Epub 2004/06/01. 10.1128/MCB.24.12.5595-5605.2004 15169918 PMC419896

[25] Zhu Y, Sun Y, Mao XO, Jin KL, Greenberg DA. Expression of poly(C)-binding proteins is differentially regulated by hypoxia and ischemia in cortical neurons. Neuroscience. 2002;110(2):191–8. Epub 2002/04/18. . 11958862 10.1016/s0306-4522(01)00522-x

[26] Huot J, Houle F, Marceau F, Landry J. Oxidative stress-induced actin reorganization mediated by the p38 mitogen-activated protein kinase/heat shock protein 27 pathway in vascular endothelial cells. Circulation research. 1997;80(3):383–92. Epub 1997/03/01. . 9048659 10.1161/01.res.80.3.383

[27] Finkel T, Holbrook NJ. Oxidants, oxidative stress and the biology of ageing. Nature. 2000;408(6809):239–47. Epub 2000/11/23. 10.1038/35041687 . 11089981

[28] Liao DF, Jin ZG, Baas AS, Daum G, Gygi SP, Aebersold R, et al Purification and identification of secreted oxidative stress-induced factors from vascular smooth muscle cells. The Journal of biological chemistry. 2000;275(1):189–96. Epub 2000/01/05. . 10617604 10.1074/jbc.275.1.189

[29] Romisch K. Endoplasmic reticulum-associated degradation. Annual review of cell and developmental biology. 2005;21:435–56. Epub 2005/10/11. 10.1146/annurev.cellbio.21.012704.133250 . 16212502

[30] Liu B, Yang Y, Qiu Z, Staron M, Hong F, Li Y, et al Folding of Toll-like receptors by the HSP90 paralogue gp96 requires a substrate-specific cochaperone. Nature communications. 2010;1:79 Epub 2010/09/25. 10.1038/ncomms1070 20865800 PMC2982182

[31] Caramalho I, Lopes-Carvalho T, Ostler D, Zelenay S, Haury M, Demengeot J. Regulatory T cells selectively express toll-like receptors and are activated by lipopolysaccharide. The Journal of experimental medicine. 2003;197(4):403–11. Epub 2003/02/20. 10.1084/jem.20021633 12591899 PMC2193858

[32] Edwards M, Zwolak A, Schafer DA, Sept D, Dominguez R, Cooper JA. Capping protein regulators fine-tune actin assembly dynamics. Nature reviews Molecular cell biology. 2014;15(10):677–89. Epub 2014/09/11. 10.1038/nrm3869 25207437 PMC4271544

[33] Papa I, Astier C, Kwiatek O, Raynaud F, Bonnal C, Lebart MC, et al Alpha actinin-CapZ, an anchoring complex for thin filaments in Z-line. Journal of muscle research and cell motility. 1999;20(2):187–97. Epub 1999/07/21. . 10412090 10.1023/a:1005489319058

[34] Bertling E, Hotulainen P, Mattila PK, Matilainen T, Salminen M, Lappalainen P. Cyclase-associated protein 1 (CAP1) promotes cofilin-induced actin dynamics in mammalian nonmuscle cells. Molecular biology of the cell. 2004;15(5):2324–34. Epub 2004/03/09. 10.1091/mbc.E04-01-0048 15004221 PMC404026

[35] Favoreel HW, Enquist LW, Feierbach B. Actin and Rho GTPases in herpesvirus biology. Trends in microbiology. 2007;15(9):426–33. Epub 2007/09/04. 10.1016/j.tim.2007.08.003 . 17764949

[36] Nadeau K, Das A, Walsh CT. Hsp90 chaperonins possess ATPase activity and bind heat shock transcription factors and peptidyl prolyl isomerases. The Journal of biological chemistry. 1993;268(2):1479–87. Epub 1993/01/15. . 8419347

[37] Eytan E, Ganoth D, Armon T, Hershko A. ATP-dependent incorporation of 20S protease into the 26S complex that degrades proteins conjugated to ubiquitin. Proceedings of the National Academy of Sciences of the United States of America. 1989;86(20):7751–5. Epub 1989/10/01. 2554287 10.1073/pnas.86.20.7751 PMC298148

[38] Zhang L, Ashendel CL, Becker GW, Morre DJ. Isolation and characterization of the principal ATPase associated with transitional endoplasmic reticulum of rat liver. The Journal of cell biology. 1994;127(6 Pt 2):1871–83. Epub 1994/12/01. 7806566 10.1083/jcb.127.6.1871 PMC2120312

[39] Horwich AL. Molecular chaperones in cellular protein folding: the birth of a field. Cell. 2014;157(2):285–8. Epub 2014/04/15. 10.1016/j.cell.2014.03.029 . 24725397

[40] Menon D, Coll R, O'Neill LA, Board PG. Glutathione transferase omega 1 is required for the lipopolysaccharide-stimulated induction of NADPH oxidase 1 and the production of reactive oxygen species in macrophages. Free radical biology & medicine. 2014;73:318–27. Epub 2014/05/31. 10.1016/j.freeradbiomed.2014.05.020 . 24873723

[41] Menon D, Coll R, O'Neill LA, Board PG. GSTO1-1 modulates metabolism in macrophages activated through the LPS and TLR4 pathway. Journal of cell science. 2015;128(10):1982–90. Epub 2015/04/25. 10.1242/jcs.167858 . 25908843

[42] Menon D, Innes A, Oakley AJ, Dahlstrom JE, Jensen LM, Brustle A, et al GSTO1-1 plays a pro-inflammatory role in models of inflammation, colitis and obesity. Scientific reports. 2017;7(1):17832 Epub 2017/12/21. 10.1038/s41598-017-17861-6 29259211 PMC5736720

[43] Garcia-Mata R, Boulter E, Burridge K. The 'invisible hand': regulation of RHO GTPases by RHOGDIs. Nature reviews Molecular cell biology. 2011;12(8):493–504. Epub 2011/07/23. 10.1038/nrm3153 21779026 PMC3260518

[44] Heath RJ, Leong JM, Visegrady B, Machesky LM, Xavier RJ. Bacterial and host determinants of MAL activation upon EPEC infection: the roles of Tir, ABRA, and FLRT3. PLoS pathogens. 2011;7(4):e1001332 Epub 2011/04/15. 10.1371/journal.ppat.1001332 21490959 PMC3072376

[45] Watanabe T, Urano E, Miyauchi K, Ichikawa R, Hamatake M, Misawa N, et al The hematopoietic cell-specific Rho GTPase inhibitor ARHGDIB/D4GDI limits HIV type 1 replication. AIDS research and human retroviruses. 2012;28(8):913–22. Epub 2011/09/23. 10.1089/AID.2011.0180 . 21936715

[46] Prehaud C, Megret F, Lafage M, Lafon M. Virus infection switches TLR-3-positive human neurons to become strong producers of beta interferon. Journal of virology. 2005;79(20):12893–904. Epub 2005/09/29. 10.1128/JVI.79.20.12893-12904.2005 16188991 PMC1235836

[47] Kent JR, Fraser NW. The cellular response to herpes simplex virus type 1 (HSV-1) during latency and reactivation. Journal of neurovirology. 2005;11(4):376–83. Epub 2005/09/16. 10.1080/13550280591002405 . 16162480

[48] Szpara ML, Kobiler O, Enquist LW. A common neuronal response to alphaherpesvirus infection. Journal of neuroimmune pharmacology: the official journal of the Society on NeuroImmune Pharmacology. 2010;5(3):418–27. Epub 2010/04/20. 10.1007/s11481-010-9212-0 20401540 PMC2990883

[49] Venkatasubramanian S, Dhiman R, Paidipally P, Cheekatla SS, Tripathi D, Welch E, et al A rho GDP dissociation inhibitor produced by apoptotic T-cells inhibits growth of Mycobacterium tuberculosis. PLoS pathogens. 2015;11(2):e1004617 Epub 2015/02/07. 10.1371/journal.ppat.1004617 25659138 PMC4450061

[50] Aitken A. 14-3-3 proteins: a historic overview. Seminars in cancer biology. 2006;16(3):162–72. Epub 2006/05/09. 10.1016/j.semcancer.2006.03.005 . 16678438

[51] Pei Z, Harrison MS, Schmitt AP. Parainfluenza virus 5 m protein interaction with host protein 14-3-3 negatively affects virus particle formation. Journal of virology. 2011;85(5):2050–9. Epub 2010/12/15. 10.1128/JVI.02111-10 21147917 PMC3067794

[52] Kenny EF, O'Neill LA. Signalling adaptors used by Toll-like receptors: an update. Cytokine. 2008;43(3):342–9. Epub 2008/08/19. 10.1016/j.cyto.2008.07.010 . 18706831

[53] Ohman T, Lietzen N, Valimaki E, Melchjorsen J, Matikainen S, Nyman TA. Cytosolic RNA recognition pathway activates 14-3-3 protein mediated signaling and caspase-dependent disruption of cytokeratin network in human keratinocytes. Journal of proteome research. 2010;9(3):1549–64. Epub 2010/01/15. 10.1021/pr901040u . 20070120

[54] Schuster TB, Costina V, Findeisen P, Neumaier M, Ahmad-Nejad P. Identification and functional characterization of 14-3-3 in TLR2 signaling. Journal of proteome research. 2011;10(10):4661–70. Epub 2011/08/11. 10.1021/pr200461p . 21827211

[55] Han S, Liang CP, DeVries-Seimon T, Ranalletta M, Welch CL, Collins-Fletcher K, et al Macrophage insulin receptor deficiency increases ER stress-induced apoptosis and necrotic core formation in advanced atherosclerotic lesions. Cell metabolism. 2006;3(4):257–66. Epub 2006/04/04. 10.1016/j.cmet.2006.02.008 . 16581003

[56] Troutman TD, Bazan JF, Pasare C. Toll-like receptors, signaling adapters and regulation of the pro-inflammatory response by PI3K. Cell cycle (Georgetown, Tex). 2012;11(19):3559–67. Epub 2012/08/17. 10.4161/cc.21572 22895011 PMC3478307

[57] Fukao T, Koyasu S. PI3K and negative regulation of TLR signaling. Trends in immunology. 2003;24(7):358–63. Epub 2003/07/16. . 12860525 10.1016/s1471-4906(03)00139-x

[58] Lopez-Pelaez M, Soria-Castro I, Bosca L, Fernandez M, Alemany S. Cot/tpl2 activity is required for TLR-induced activation of the Akt p70 S6k pathway in macrophages: Implications for NO synthase 2 expression. European journal of immunology. 2011;41(6):1733–41. Epub 2011/04/07. 10.1002/eji.201041101 . 21469113

[59] Luyendyk JP, Schabbauer GA, Tencati M, Holscher T, Pawlinski R, Mackman N. Genetic analysis of the role of the PI3K-Akt pathway in lipopolysaccharide-induced cytokine and tissue factor gene expression in monocytes/macrophages. Journal of immunology (Baltimore, Md: 1950). 2008;180(6):4218–26. Epub 2008/03/07. 18322234 10.4049/jimmunol.180.6.4218 PMC2834303

[60] Fordham JB, Naqvi AR, Nares S. miR-24 Regulates Macrophage Polarization and Plasticity. Journal of clinical & cellular immunology. 2015;6(5). Epub 2016/01/26. 10.4172/2155-9899.1000362 26807309 PMC4721581

[61] Lee JS, Nauseef WM, Moeenrezakhanlou A, Sly LM, Noubir S, Leidal KG, et al Monocyte p110alpha phosphatidylinositol 3-kinase regulates phagocytosis, the phagocyte oxidase, and cytokine production. Journal of leukocyte biology. 2007;81(6):1548–61. Epub 2007/03/21. 10.1189/jlb.0906564 . 17369495

[62] Matta SK, Kumar D. AKT mediated glycolytic shift regulates autophagy in classically activated macrophages. The international journal of biochemistry & cell biology. 2015;66:121–33. Epub 2015/07/30. 10.1016/j.biocel.2015.07.010 . 26222186

[63] Covarrubias AJ, Aksoylar HI, Horng T. Control of macrophage metabolism and activation by mTOR and Akt signaling. Seminars in immunology. 2015;27(4):286–96. Epub 2015/09/12. 10.1016/j.smim.2015.08.001 26360589 PMC4682888

[64] Arranz A, Doxaki C, Vergadi E, Martinez de la Torre Y, Vaporidi K, Lagoudaki ED, et al Akt1 and Akt2 protein kinases differentially contribute to macrophage polarization. Proceedings of the National Academy of Sciences of the United States of America. 2012;109(24):9517–22. Epub 2012/06/01. 10.1073/pnas.1119038109 22647600 PMC3386059

[65] Ruckerl D, Jenkins SJ, Laqtom NN, Gallagher IJ, Sutherland TE, Duncan S, et al Induction of IL-4Ralpha-dependent microRNAs identifies PI3K/Akt signaling as essential for IL-4-driven murine macrophage proliferation in vivo. Blood. 2012;120(11):2307–16. Epub 2012/08/03. 10.1182/blood-2012-02-408252 22855601 PMC3501641

[66] Happel N, Doenecke D. Histone H1 and its isoforms: contribution to chromatin structure and function. Gene. 2009;431(1–2):1–12. Epub 2008/12/09. 10.1016/j.gene.2008.11.003 . 19059319

[67] Fasching L, Kapopoulou A, Sachdeva R, Petri R, Jonsson ME, Manne C, et al TRIM28 represses transcription of endogenous retroviruses in neural progenitor cells. Cell reports. 2015;10(1):20–8. Epub 2014/12/30. 10.1016/j.celrep.2014.12.004 25543143 PMC4434221

[68] Hardin M, Silverman EK. Chronic Obstructive Pulmonary Disease Genetics: A Review of the Past and a Look Into the Future. Chronic obstructive pulmonary diseases (Miami, Fla). 2014;1(1):33–46. Epub 2014/05/06. 10.15326/jcopdf.1.1.2014.0120 28848809 PMC5559139

[69] Zhang Z, Zheng F, Yu Z, Hao J, Chen M, Yu W, et al XRCC5 cooperates with p300 to promote cyclooxygenase-2 expression and tumor growth in colon cancers. PloS one. 2017;12(10):e0186900 Epub 2017/10/20. 10.1371/journal.pone.0186900 29049411 PMC5648251

[70] Jahantigh D, Salimi S, Mousavi M, Moossavi M, Mohammadoo-Khorasani M, Narooei-nejad M, et al Association Between Functional Polymorphisms of DNA Double-Strand Breaks in Repair Genes XRCC5, XRCC6 and XRCC7 with the Risk of Systemic Lupus Erythematosus in South East Iran. DNA and cell biology. 2015;34(5):360–6. Epub 2015/03/11. 10.1089/dna.2014.2465 . 25756210

[71] Stark JM, Pierce AJ, Oh J, Pastink A, Jasin M. Genetic steps of mammalian homologous repair with distinct mutagenic consequences. Molecular and cellular biology. 2004;24(21):9305–16. Epub 2004/10/16. 10.1128/MCB.24.21.9305-9316.2004 15485900 PMC522275

[72] Zhu Y, Tomlinson RL, Lukowiak AA, Terns RM, Terns MP. Telomerase RNA accumulates in Cajal bodies in human cancer cells. Molecular biology of the cell. 2004;15(1):81–90. Epub 2003/10/07. 10.1091/mbc.E03-07-0525 14528011 PMC307529

[73] Wang Q, Sawyer IA, Sung MH, Sturgill D, Shevtsov SP, Pegoraro G, et al Cajal bodies are linked to genome conformation. Nature communications. 2016;7:10966 Epub 2016/03/22. 10.1038/ncomms10966 26997247 PMC4802181

[74] Gong J, Li Y, Liu CJ, Xiang Y, Li C, Ye Y, et al A Pan-cancer Analysis of the Expression and Clinical Relevance of Small Nucleolar RNAs in Human Cancer. Cell reports. 2017;21(7):1968–81. Epub 2017/11/16. 10.1016/j.celrep.2017.10.070 . 29141226

