# Supplementary figures and images for: Comparative proteomic study reveals the enhanced immune response with the blockade of interleukin 10 with anti-IL-10 and anti-IL-10 receptor antibodies in human U937 cells
Source: PLoS One. 2019 Mar 21;14(3):e0213813. doi: 10.1371/journal.pone.0213813 (PMC6428271; doi:10.1371/journal.pone.0213813)

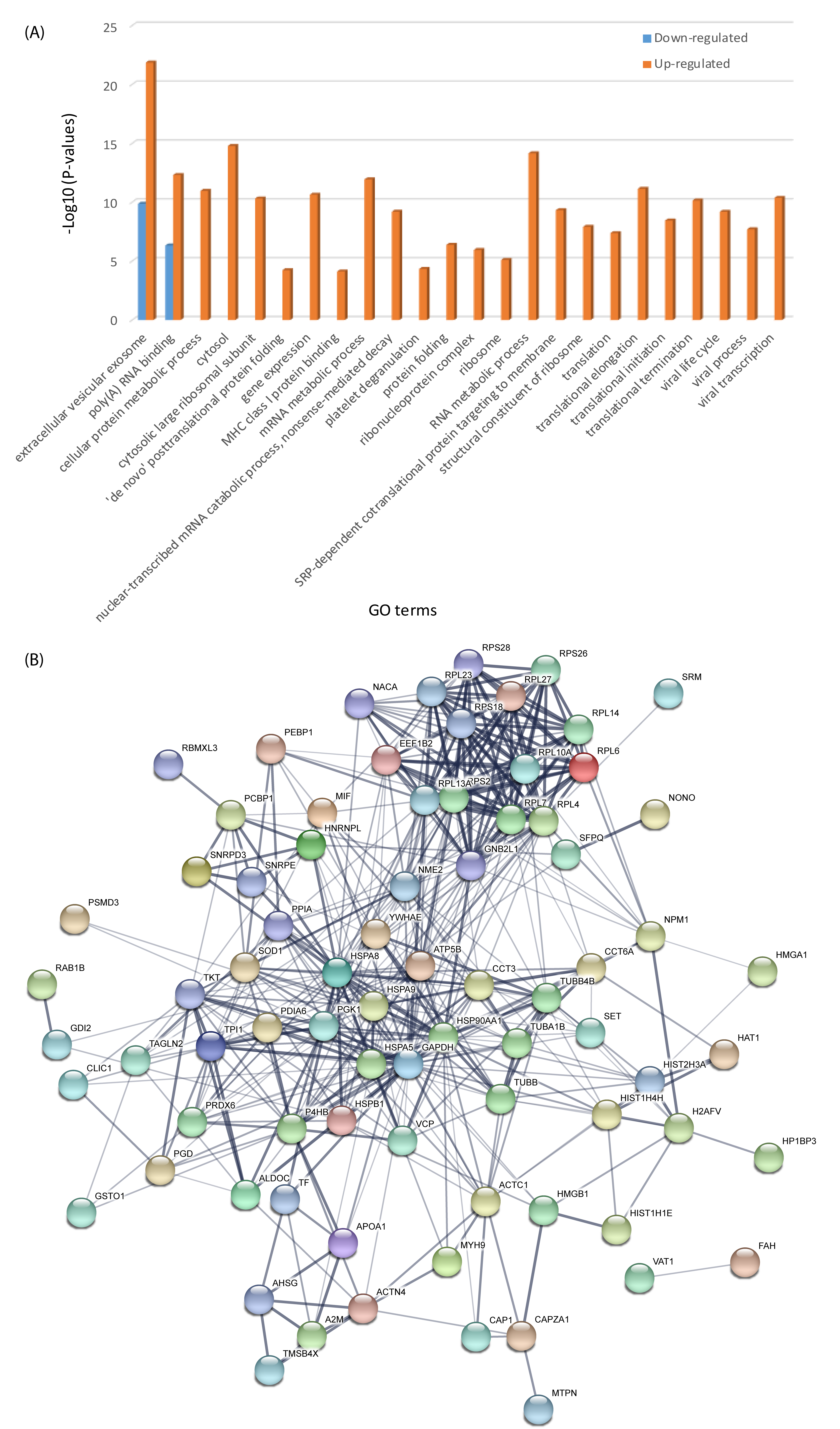

Supplement: S3 Fig — (TIF) [file pone.0213813.s003.tif]

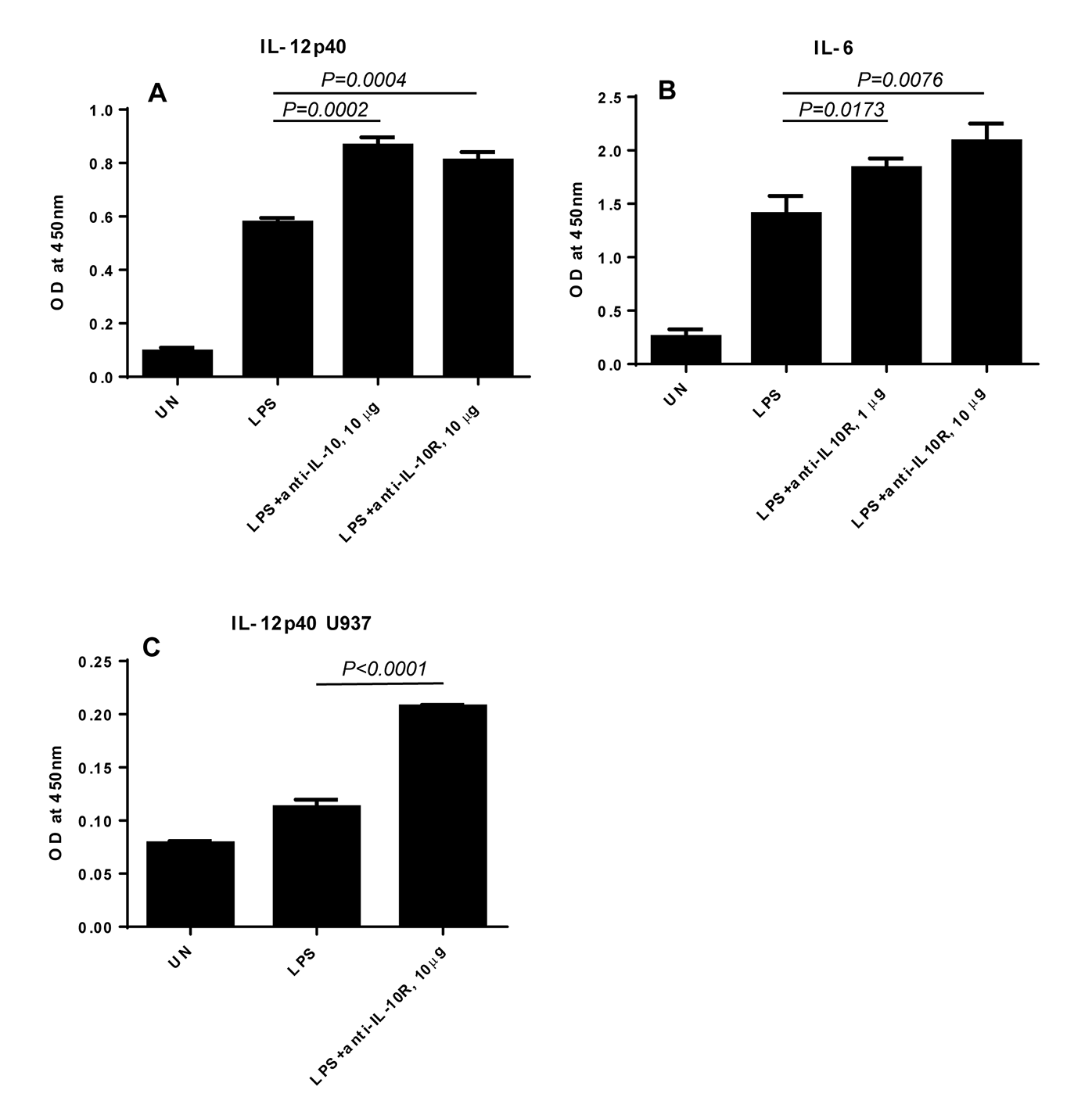

Supplement: S4 Fig — 5×10^5^ of mouse PBMCs (A and B) or 5×10^5^ U937 cells (C) were either left untreated (UN) or stimulated with 100 ng of LPS, in the presence or absence of anti-IL-10 or anti-IL10R antibodies and cultured overnight. Supernatants were collected and measured by IL-6 or IL-12p40 ELISA kit from eBioscience (San Diego, CA, USA). Statistical analysis was performed by the two-tailed t-test. (TIF) [file pone.0213813.s004.tif]
