# Supplementary material for: Comparative proteomic study reveals the enhanced immune response with the blockade of interleukin 10 with anti-IL-10 and anti-IL-10 receptor antibodies in human U937 cells
Source: PLoS One. 2019 Mar 21;14(3):e0213813. doi: 10.1371/journal.pone.0213813 (PMC6428271; doi:10.1371/journal.pone.0213813)
Supplement: S1 File — (PDF) [file pone.0213813.s009.pdf]

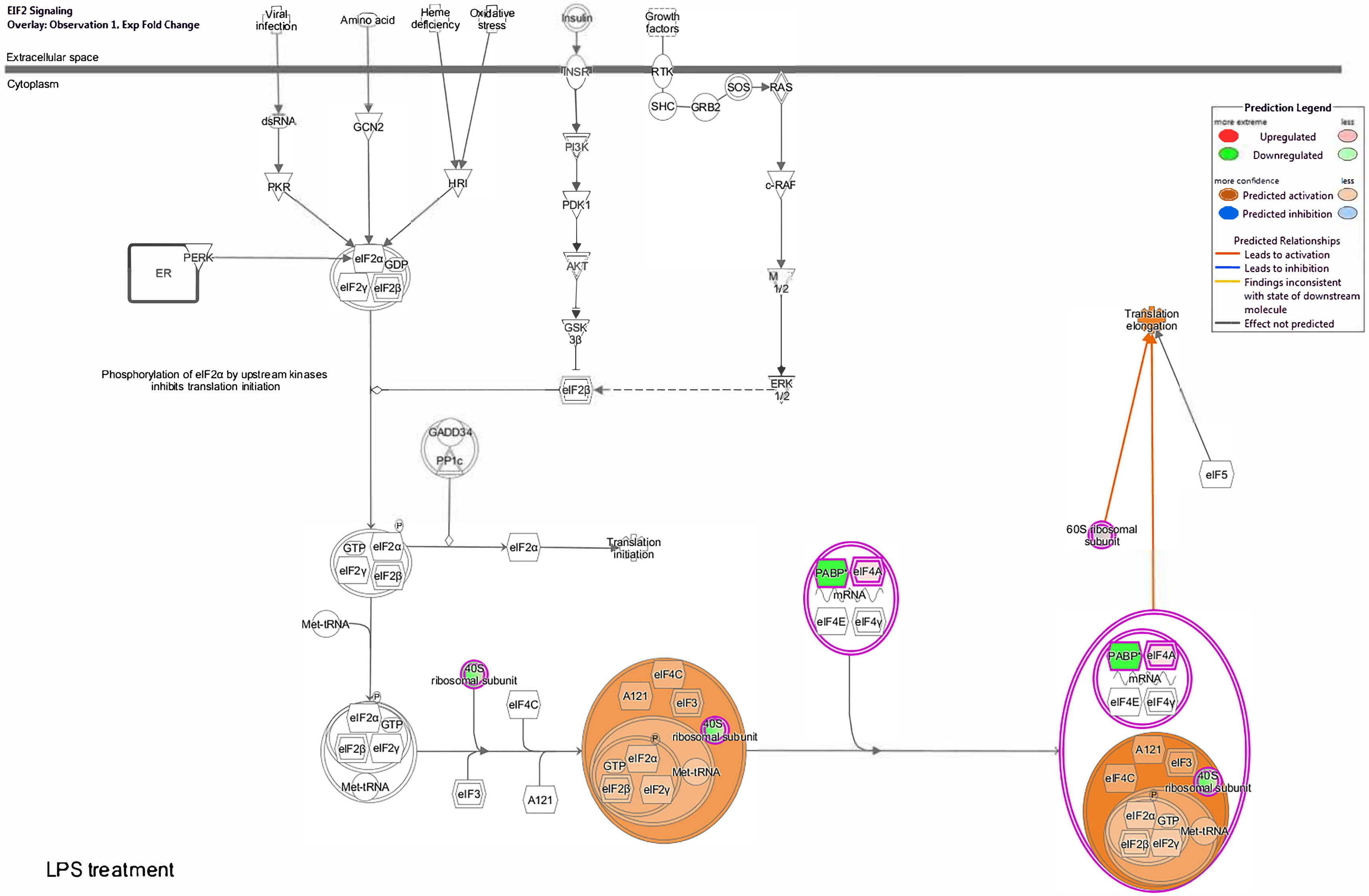

LPS treatment

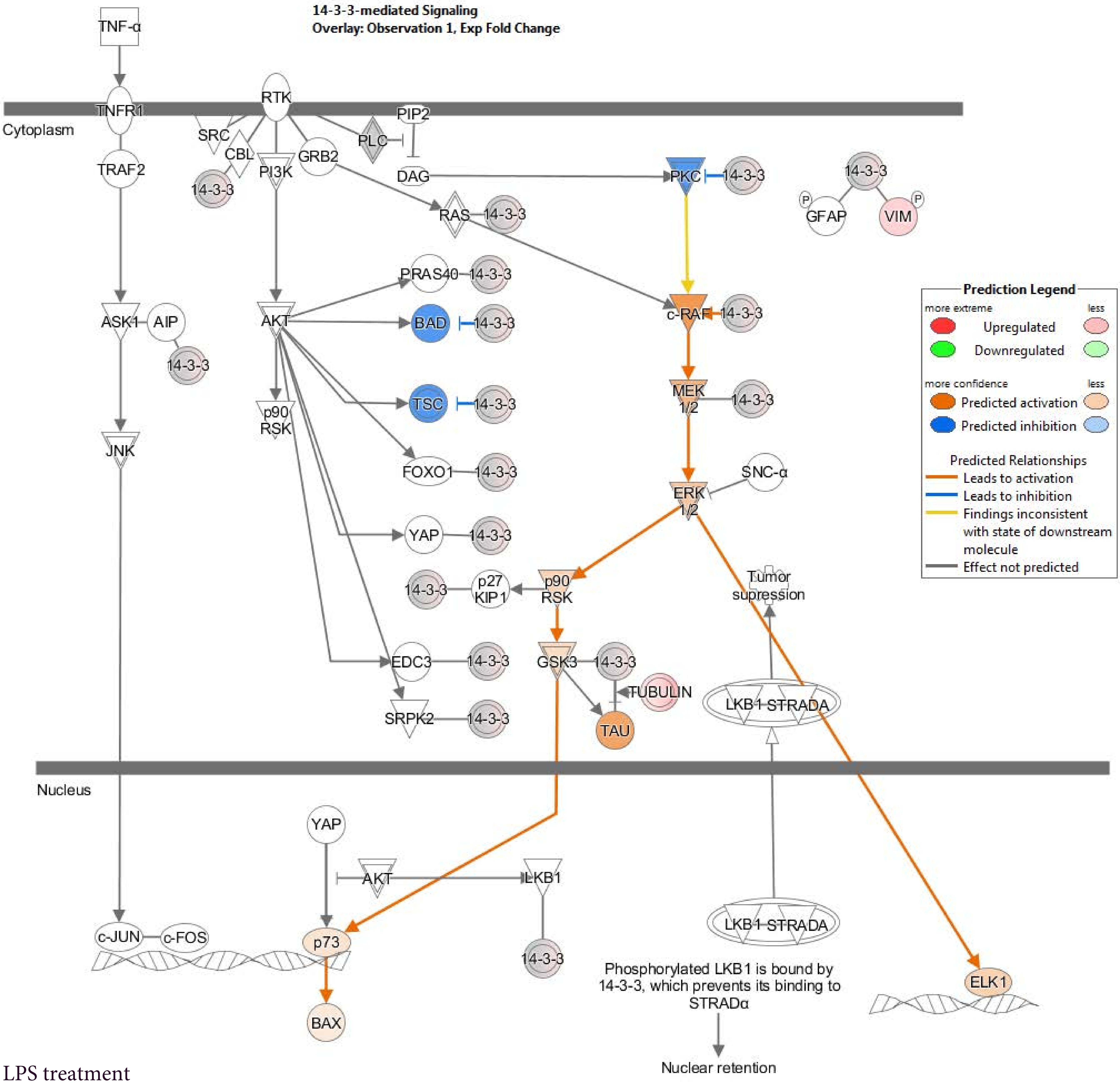

LPS treatment

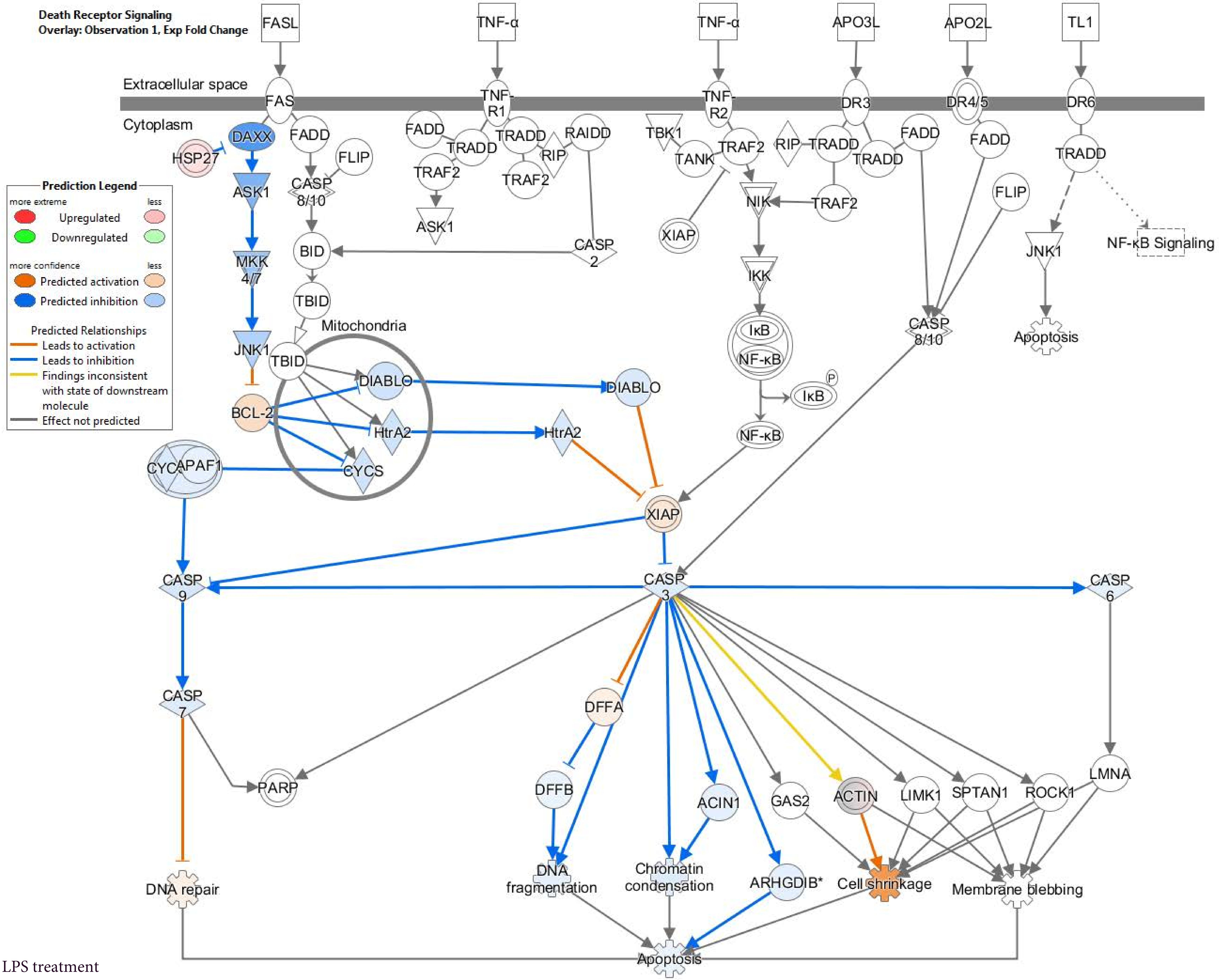

LPS treatment

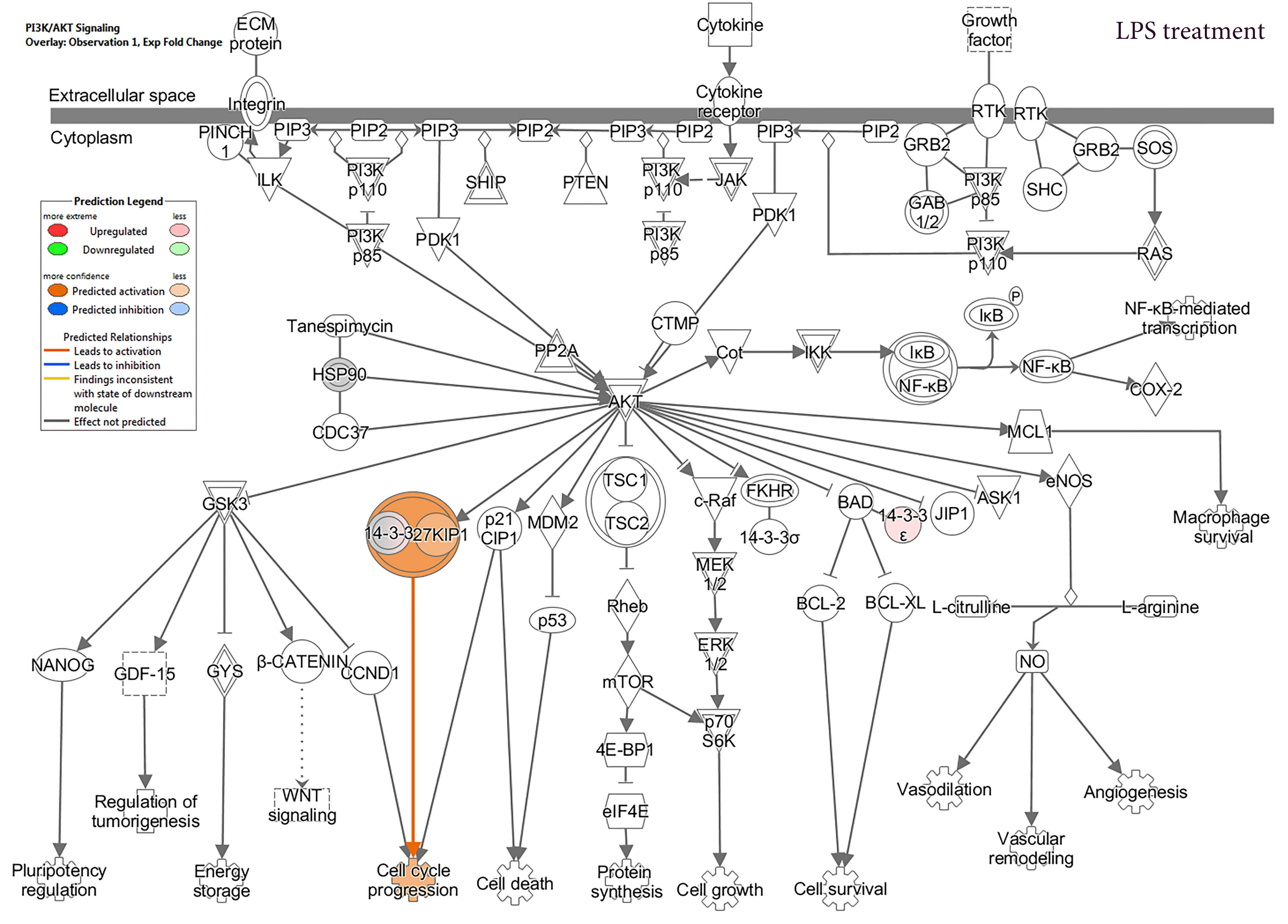
PI3K/AKT Signaling
Overlay: Observation 1, Exp Fold Change
LPS treatment
ECM protein
Cytokine
Growth factor
Extracellular space
Cytoplasm
Integrin
Cytokine receptor
RTK
RTK
PINCH 1
PIP3
PIP2
PIP3
PIP2
PIP3
PIP2
PIP3
PIP2
GRB2
GRB2
SOS
ILK
PI3K p110
SHIP
PTEN
PI3K p110
JAK
PI3K p85
SHC
GAB 1/2
PI3K p85
PDK1
PDK1
PI3K p85
PI3K p110
RAS
Prediction Legend
more extreme
less
Upregulated
Downregulated
more confidence
less
Predicted activation
Predicted inhibition
Predicted Relationships
Leads to activation
Leads to inhibition
Findings inconsistent with state of downstream molecule
Effect not predicted
Tanespimycin
HSP90
CDC37
PP2A
CTMP
AKT
Cot
IKK
IκB
NF-κB
IκB
P
NF-κB
NF-κB-mediated transcription
COX-2
MCL1
eNOS
Macrophage survival
GSK3
14-3-3
27KIP1
p21 CIP1
MDM2
TSC1
TSC2
c-Raf
FKHR
14-3-3σ
BAD
14-3-3 ε
JIP1
ASK1
MEK 1/2
ERK 1/2
p53
Rheb
mTOR
4E-BP1
eIF4E
p70 S6K
BCL-2
BCL-XL
L-citrulline
L-arginine
NO
NANOG
GDF-15
GYS
β-CATENIN
CCND1
Regulation of tumorigenesis
WNT signaling
Pluripotency regulation
Energy storage
Cell cycle progression
Cell death
Protein synthesis
Cell growth
Cell survival
Vasodilation
Vascular remodeling
Angiogenesis

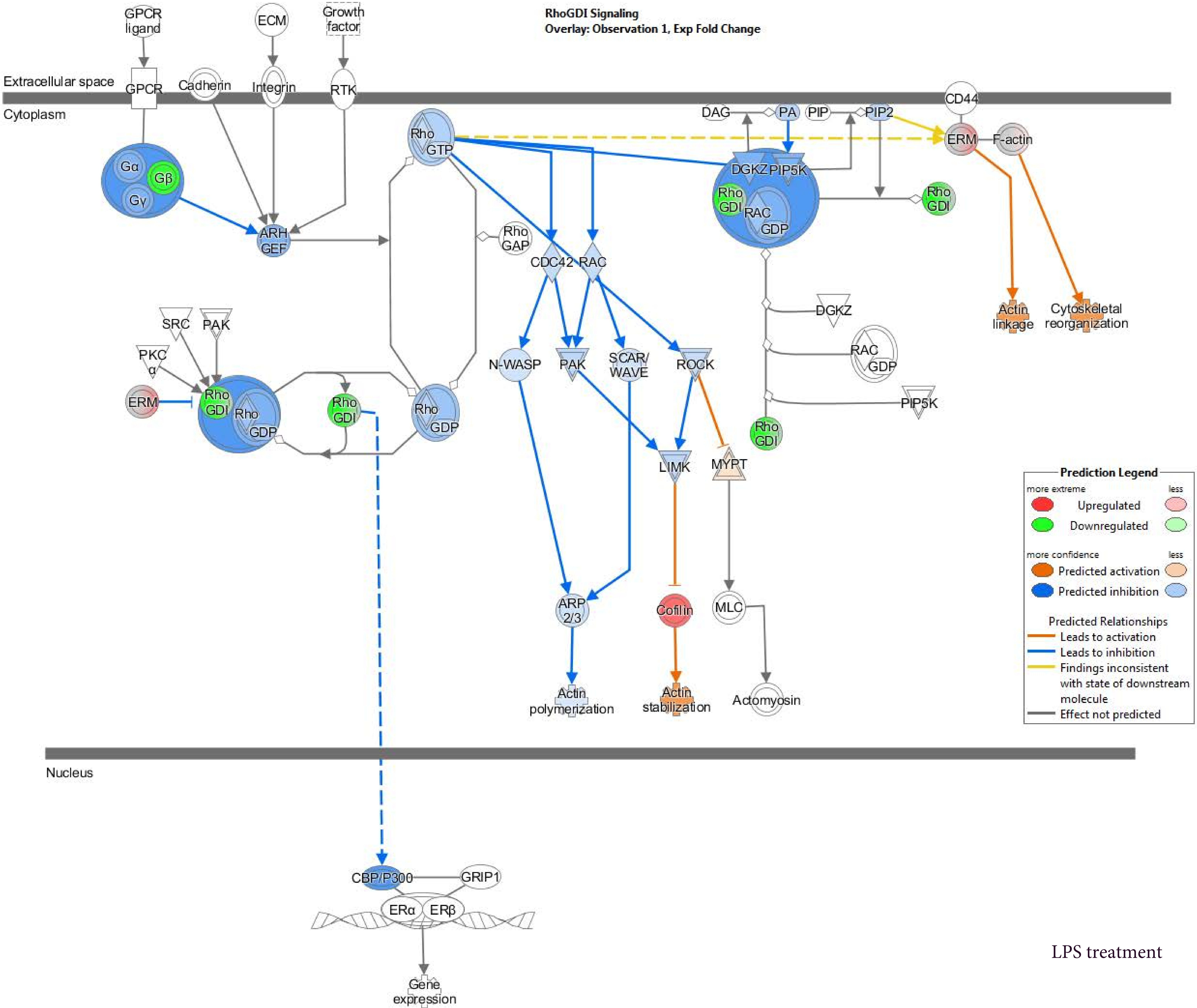

LPS treatment

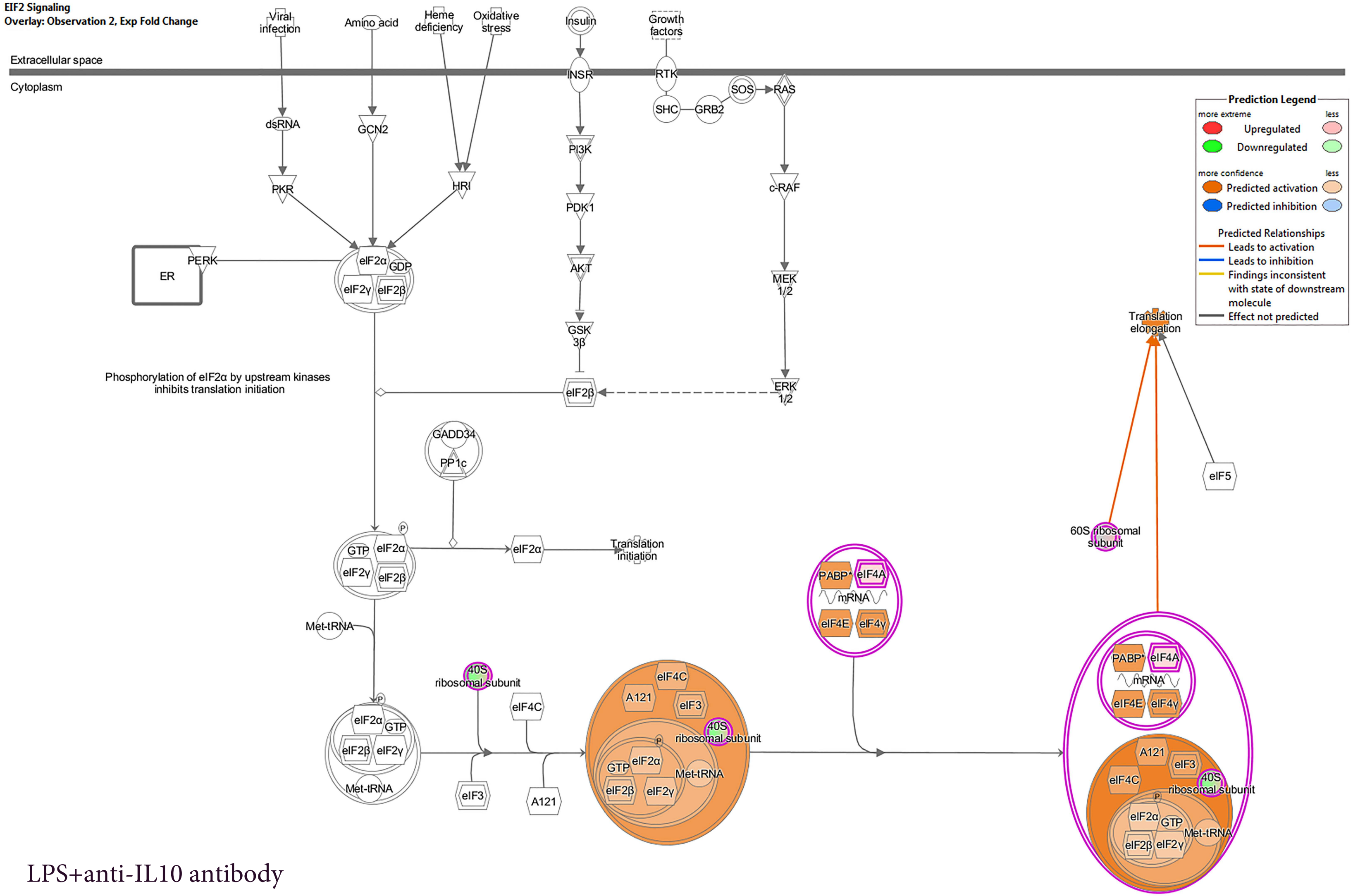

LPS+anti-IL10 antibody

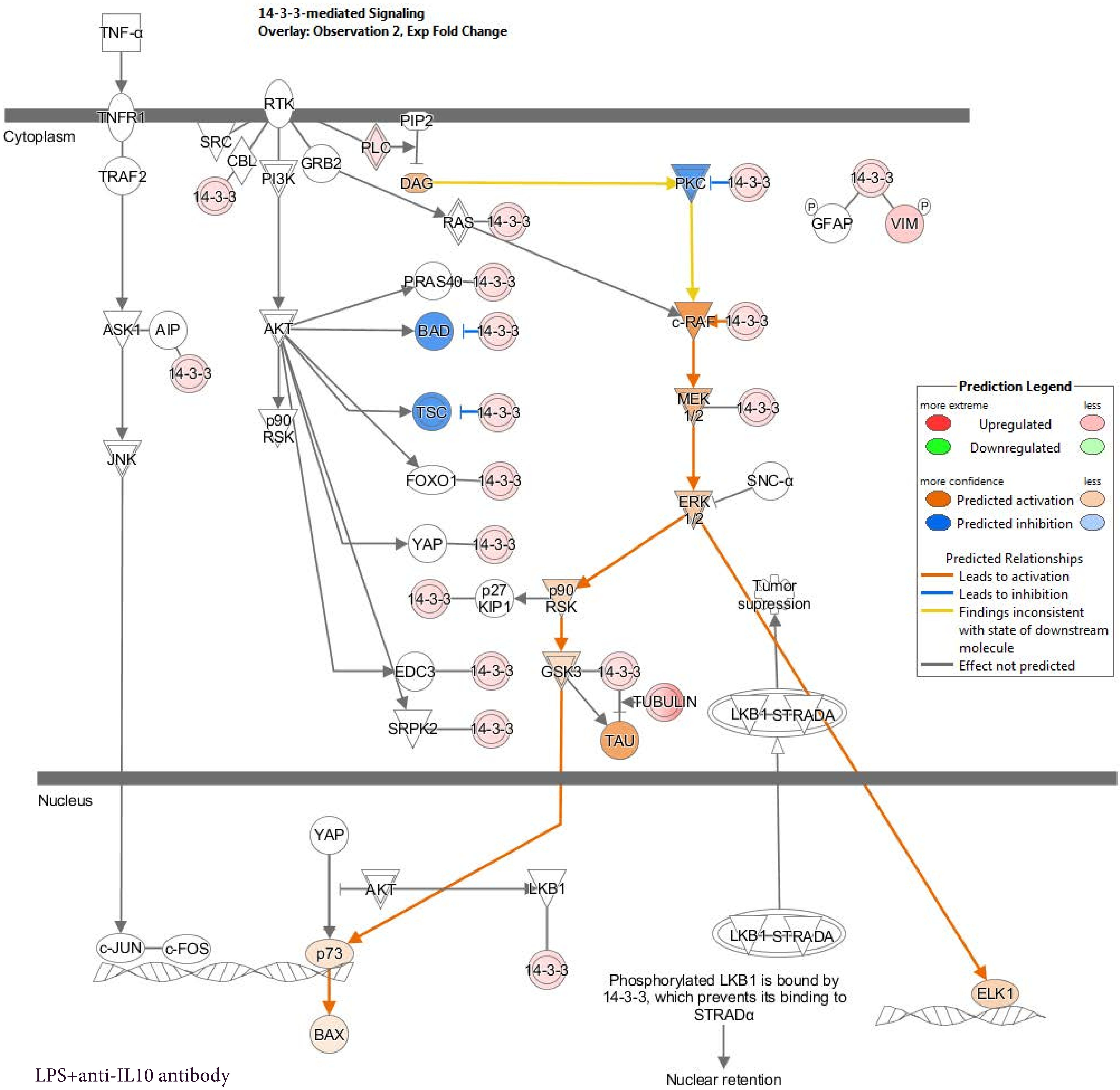

LPS+anti-IL10 antibody

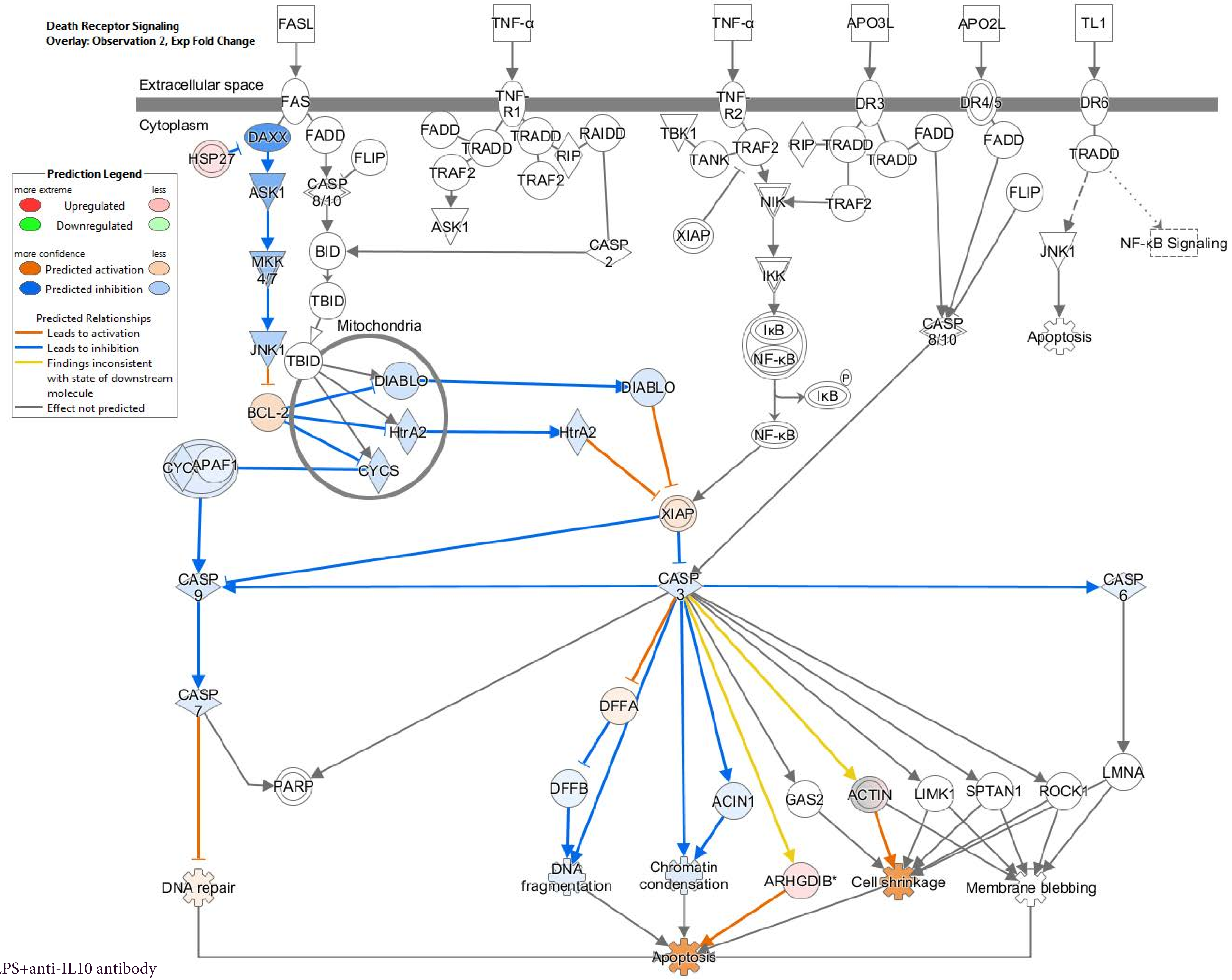

LPS+anti-IL10 antibody

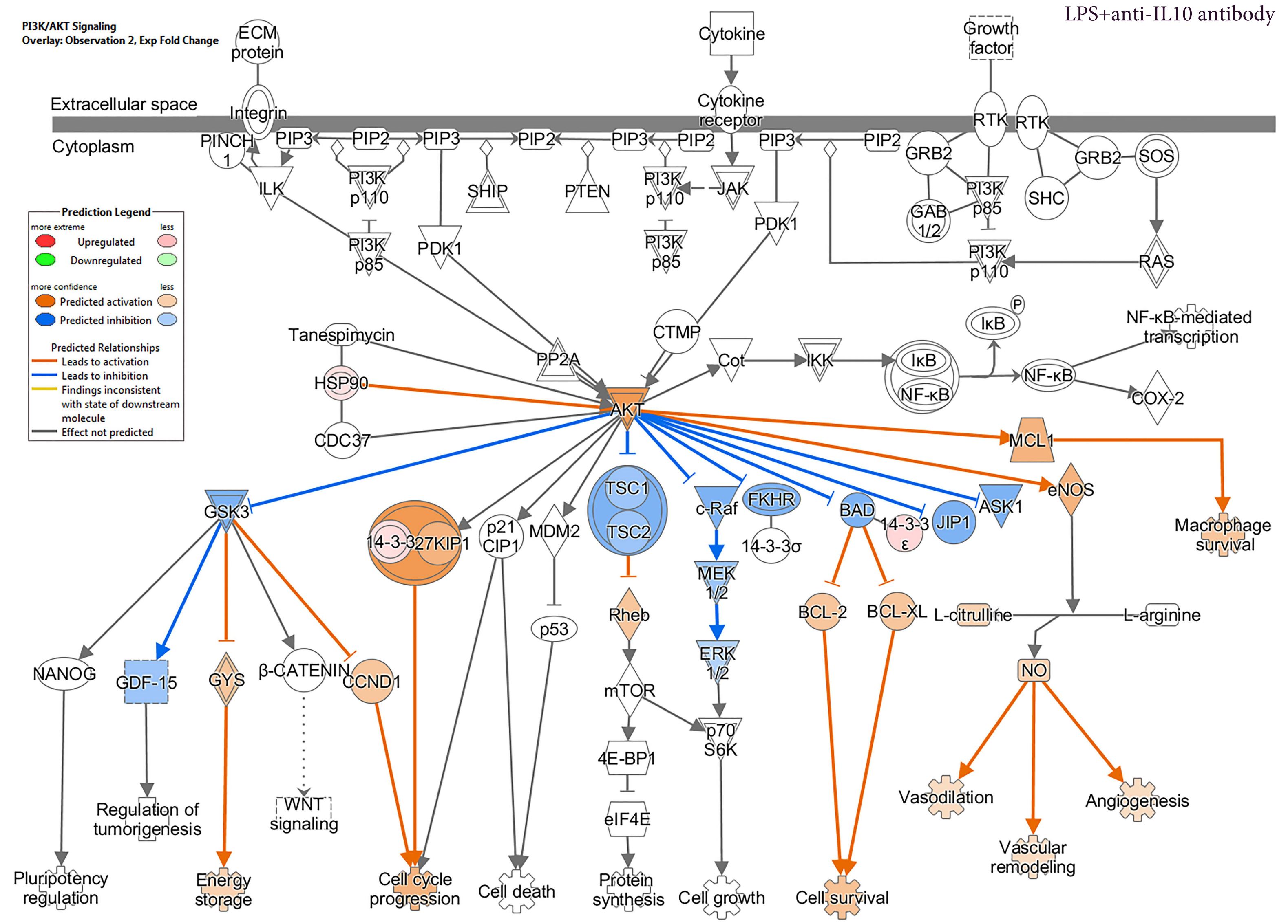
LPS+anti-IL10 antibody
PI3K/AKT Signaling
Overlay: Observation 2, Exp Fold Change
Extracellular space
Cytoplasm
ECM protein
Integrin
Cytokine
Cytokine receptor
Growth factor
RTK
RTK
PINCH 1
ILK
PIP3
PIP2
PIP3
PIP2
PIP3
PIP2
PIP3
PIP2
PI3K p110
PI3K p85
PDK1
SHIP
PTEN
PI3K p110
PI3K p85
JAK
PDK1
GRB2
GAB 1/2
PI3K p85
PI3K p110
GRB2
SOS
SHC
RAS
Prediction Legend
more extreme
less
Upregulated
Downregulated
more confidence
less
Predicted activation
Predicted inhibition
Predicted Relationships
Leads to activation
Leads to inhibition
Findings inconsistent with state of downstream molecule
Effect not predicted
Tanespimycin
HSP90
CDC37
PP2A
CTMP
AKT
Cot
IKK
IκB
NF-κB
IκB
P
NF-κB
NF-κB-mediated transcription
COX-2
MCL1
eNOS
Macrophage survival
GSK3
14-3-3
27KIP1
p21 CIP1
MDM2
TSC1
TSC2
c-Raf
FKHR
14-3-3σ
BAD
14-3-3 ε
JIP1
ASK1
MEK 1/2
ERK 1/2
p53
Rheb
mTOR
4E-BP1
eIF4E
p70 S6K
BCL-2
BCL-XL
L-citrulline
L-arginine
NO
NANOG
GDF-15
GYS
β-CATENIN
CCND1
Regulation of tumorigenesis
WNT signaling
Pluripotency regulation
Energy storage
Cell cycle progression
Cell death
Protein synthesis
Cell growth
Cell survival
Vasodilation
Vascular remodeling
Angiogenesis

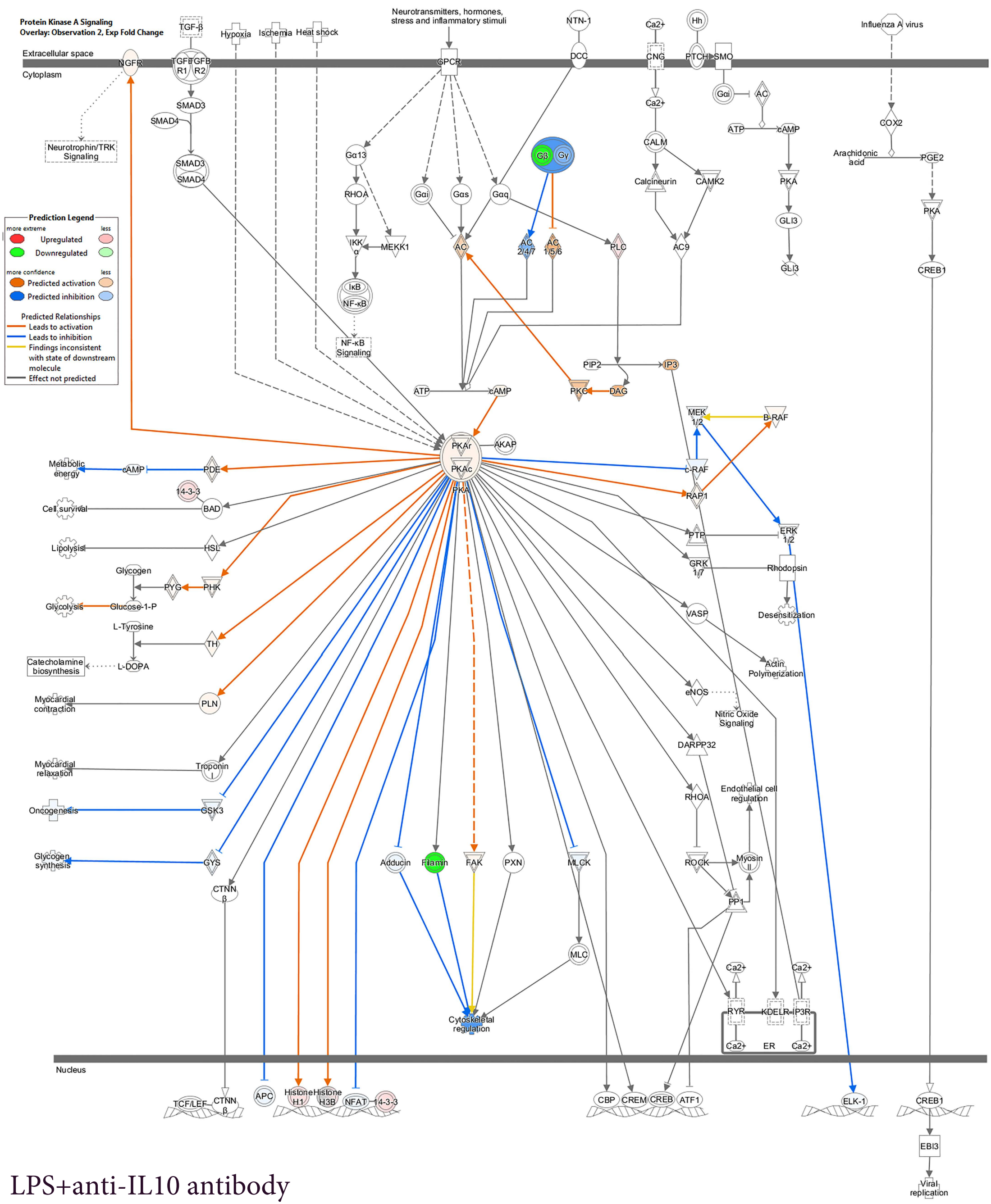

LPS+anti-IL10 antibody

LPS+anti-IL10 antibody

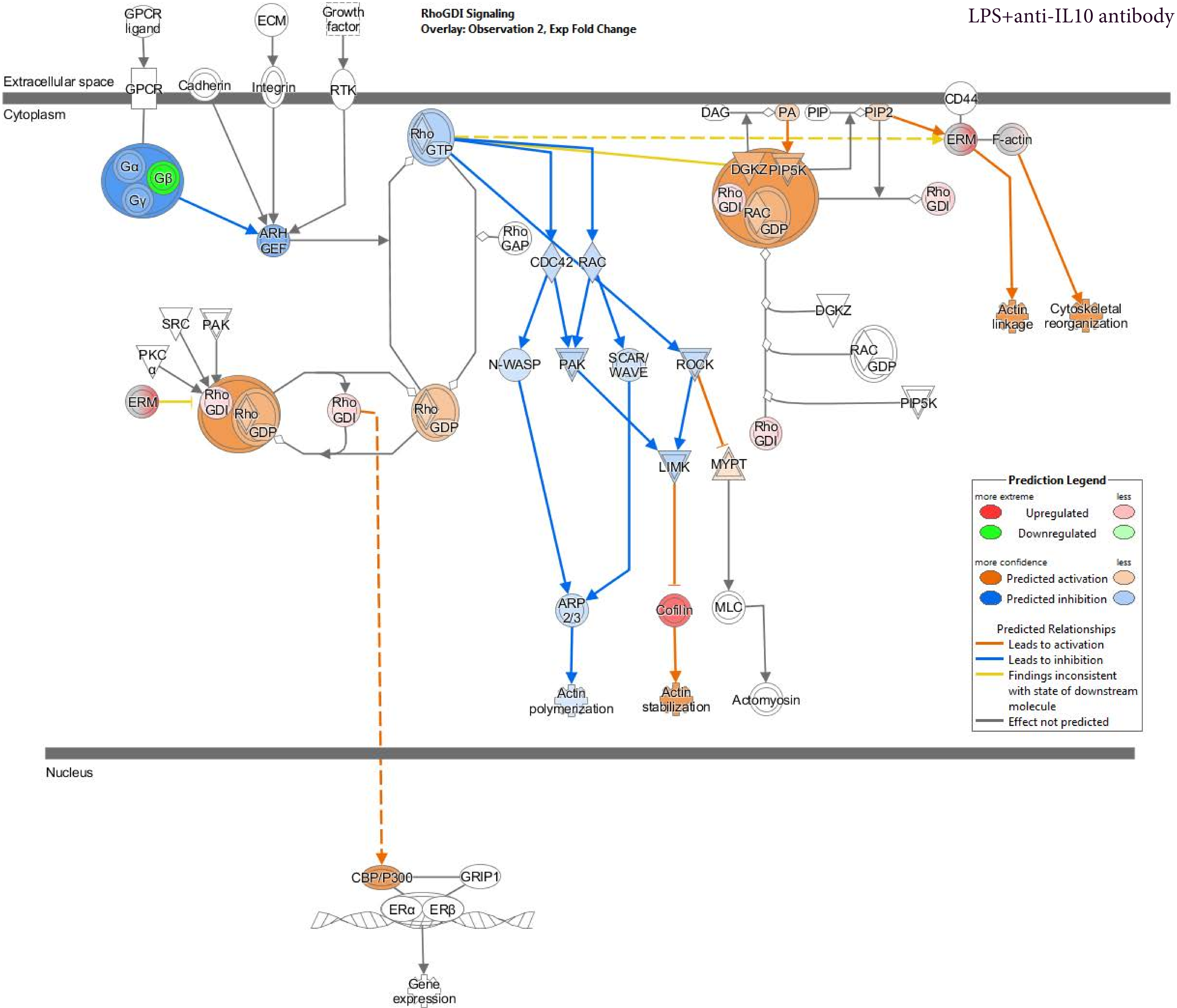

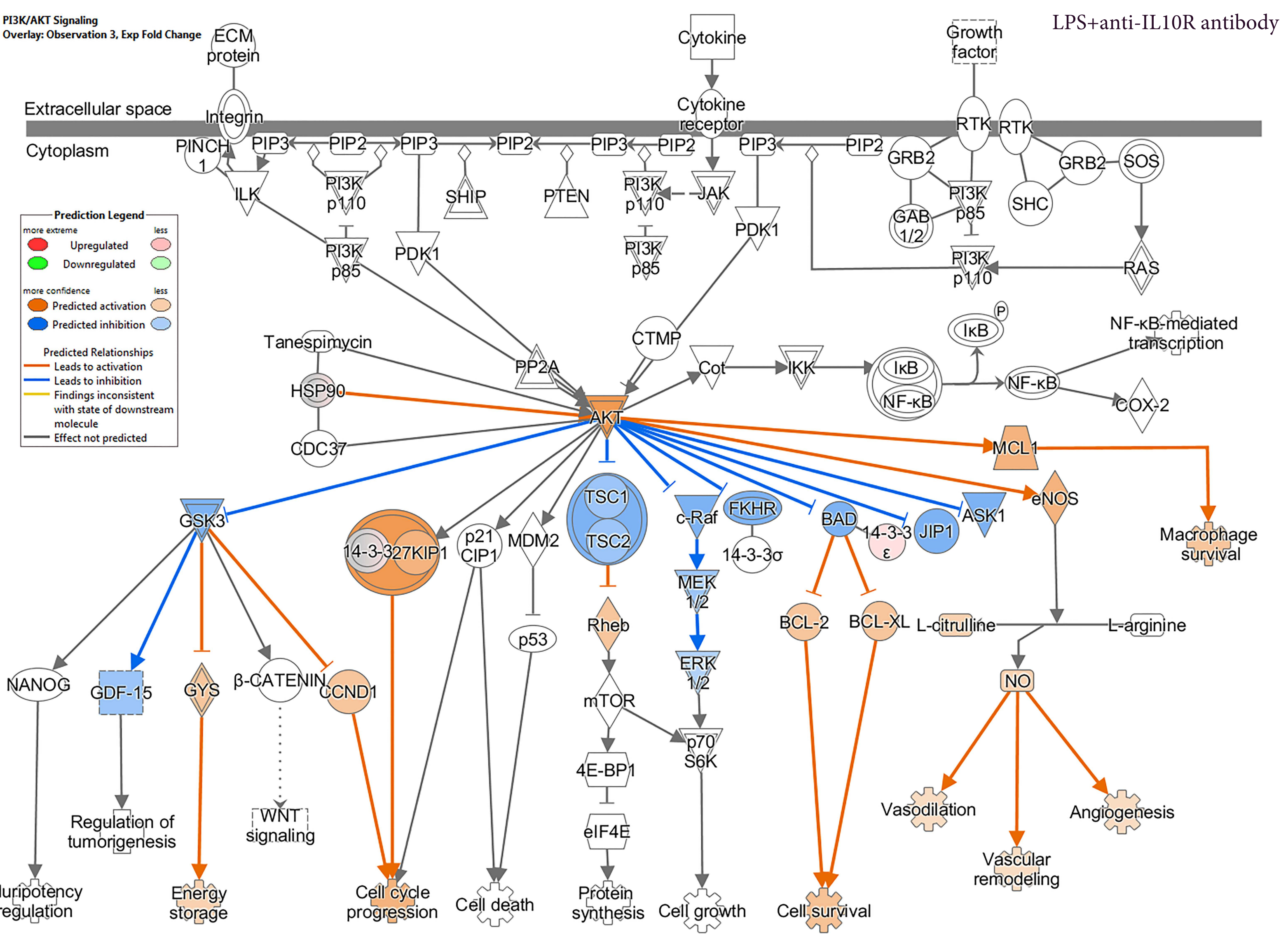
PI3K/AKT Signaling
Overlay: Observation 3, Exp Fold Change
LPS+anti-IL10R antibody
ECM protein
Cytokine
Growth factor
Extracellular space
Integrin
Cytokine receptor
RTK
RTK
Cytoplasm
PINCH 1
PIP3
PIP2
PIP3
PIP2
PIP3
PIP2
PIP3
PIP2
GRB2
GRB2
SOS
ILK
PI3K p110
SHIP
PTEN
PI3K p110
JAK
PI3K p85
SHC
GAB 1/2
Prediction Legend
more extreme
less
Upregulated
Downregulated
more confidence
less
Predicted activation
Predicted inhibition
Predicted Relationships
Leads to activation
Leads to inhibition
Findings inconsistent with state of downstream molecule
Effect not predicted
PI3K p85
PDK1
PI3K p85
PDK1
PI3K p110
RAS
IκB
P
NF-κB-mediated transcription
Tanespimycin
CTMP
PP2A
Cot
IKK
IκB
NF-κB
NF-κB
HSP90
AKT
COX-2
CDC37
MCL1
eNOS
GSK3
TSC1
TSC2
c-Raf
FKHR
BAD
14-3-3 ε
JIP1
ASK1
Macrophage survival
14-3-3
27KIP1
p21 CIP1
MDM2
14-3-3σ
MEK 1/2
Rheb
BCL-2
BCL-XL
L-citrulline
L-arginine
p53
NANOG
GDF-15
GYS
β-CATENIN
CCND1
ERK 1/2
mTOR
NO
p70 S6K
4E-BP1
eIF4E
Regulation of tumorigenesis
WNT signaling
Vasodilation
Angiogenesis
Vascular remodeling
luripotency regulation
Energy storage
Cell cycle progression
Cell death
Protein synthesis
Cell growth
Cell survival
